# Evolutionary Progress of Silica Aerogels and Their Classification Based on Composition: An Overview

**DOI:** 10.3390/nano13091498

**Published:** 2023-04-27

**Authors:** Puttavva Meti, Qi Wang, D. B. Mahadik, Kyu-Yeon Lee, Young-Dae Gong, Hyung-Ho Park

**Affiliations:** 1 Innovative Drug Library Research Center, Department of Chemistry, Dongguk University, Seoul 04620, Republic of Korea; pmeti25@gmail.com; 2 Department of Materials Science and Engineering, Yonsei University, Seoul 03722, Republic of Korea; qwang@yonsei.ac.kr (Q.W.);

**Keywords:** silica aerogels, organic-modified silica aerogels

## Abstract

Aerogels are highly porous materials with fascinating properties prepared using sol-gel chemistry. Due to their unique physical and chemical properties, aerogels are recognized as potential candidates for diverse applications, including thermal insulation, sensor, environmental remediation, etc. Despite these applications, aerogels are not routinely found in our daily life because they are fragile and have highly limited scale-up productions. It remains extremely challenging to improve the mechanical properties of aerogels without adversely affecting their other properties. To boost the practical applications, it is necessary to develop efficient, low-cost methods to produce aerogels in a sustainable way. This comprehensive review surveys the progress in the development of aerogels and their classification based on the chemical composition of the network. Recent achievements in organic, inorganic, and hybrid materials and their outstanding physical properties are discussed. The major focus of this review lies in approaches that allow tailoring of aerogel properties to meet application-driven requirements. We begin with a brief discussion of the fundamental issues in silica aerogels and then proceed to provide an overview of the synthesis of organic and hybrid aerogels from various precursors. Organic aerogels show promising results with excellent mechanical strength, but there are still several issues that need further exploration. Finally, growing points and perspectives of the aerogel field are summarized.

## 1. Introduction

**Figure 1:**
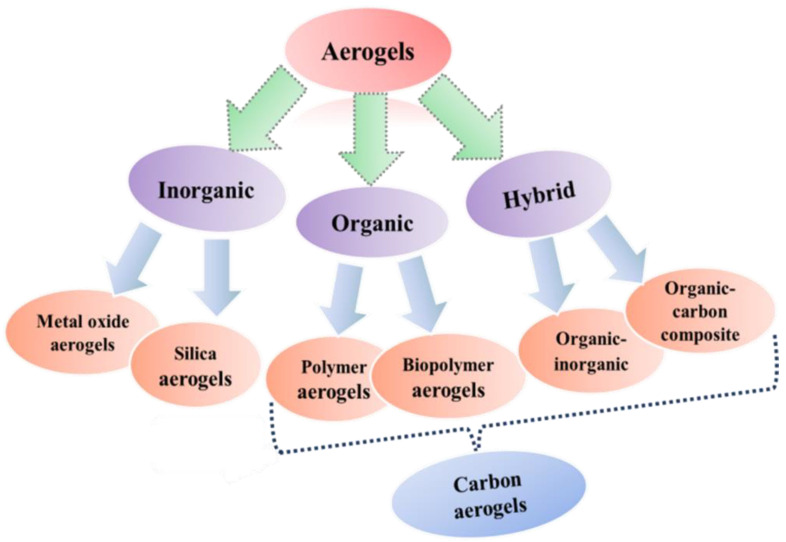
Classification of aerogels based on precursor composition.

Several approaches have been used to classify aerogels based on their material forms (monoliths, granules, powders, and films), chemical composition (organic, inorganic, and hybrid), and microstructure (microporous, mesoporous, and macroporous). Different forms, compositions, and microstructures can offer different applications for and greater functionality of aerogels. The present review provides a panorama of aerogels based on their composition. Aerogels can be essentially divided into three categories: inorganic (metal and metal oxides), organic (synthetic polymers and biopolymers), and organic–inorganic hybrid materials (Figure 1). Carbon aerogels are another important class of aerogels with different physical properties that are obtained from pyrolysis of organic and organic and inorganic composite aerogels. Carbon aerogels are characterized by excellent thermal and electrical conductivity, high porosity, and high specific surface area (>2000 m^2^/g). A good introduction to carbon-based aerogels can be found in the literature [1,2,3,4,5].

### 1.1. Inorganic Silica Aerogels

**Figure 2:**
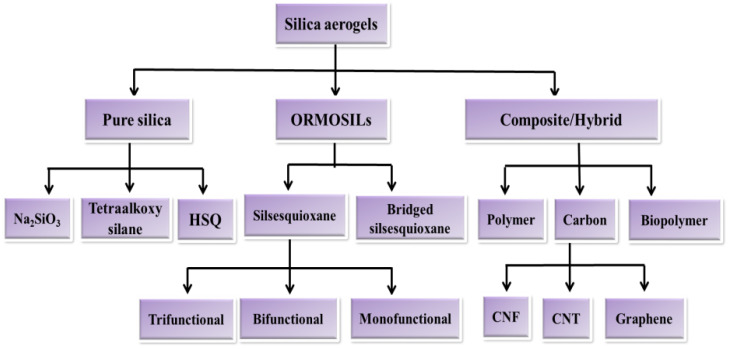
Classification of silica aerogels based on precursor composition.

Inorganic aerogels were the first widely studied aerogels and have been applied the most [6,7]. Nearly all metal alkoxides are known to form porous aerogels. Synthesis of metal oxide aerogels (titanium, zirconium, tin, aluminum, etc.) with different geometries has been comprehensively reviewed by Sui and Charpentier [8]. Among the inorganic, silica aerogels are the most widely studied commercially important class of material, and enjoy exclusive attributes such as high porosity, large surface area, and exceptionally low thermal conductivity [9,10,11]. The choice of precursors helps to control the properties of the final materials, thus leading to products with tailored physical and chemical properties. Overall, silica aerogels are divided into three categories derived from (1) pure silica, (2) organically modified silicas (ORMOSILs), and (3) organic–inorganic composite aerogels as illustrated in Figure 2.

#### 1.1.1. Sodium-Silicate-Based Aerogels

**Scheme 1:**
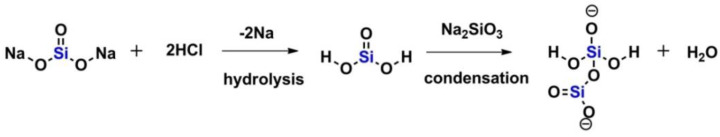
Reaction mechanisms of neutralization and condensation of sodium silicate.

Silica aerogels stand out as the most studied inorganic aerogels, although galaxy gel-forming materials are known in the literature. Silica aerogels have played a dominant role in both academics and industry since their first report in the 1930s [12]. The architecture of silica aerogels consists of a mesoporous structure with interconnected Si-O-Si bonds. Kistler demonstrated the first example of silica aerogels by treating an aqueous solution of sodium silicate (water glass) with hydrochloric acid. The reaction mechanism is illustrated in Scheme 1. The aquagel obtained from this precursor is obtained using the sol-gel transition via a simple neutralization or via a two-stage reaction, followed by a supercritical drying technique.

**Scheme 2:**
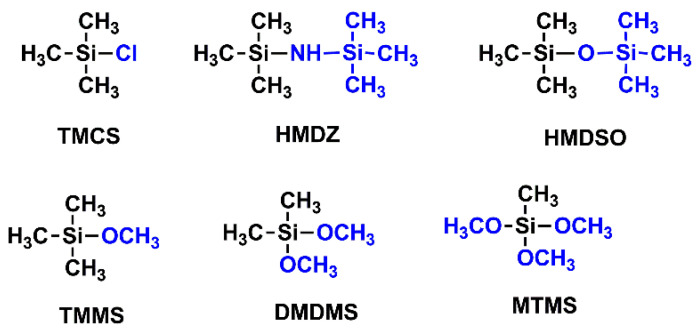
Chemical structure of silylating agents used for surface modification.

*Native silica aerogels are hygroscopic; the residual Si–OH bonds on the silica surface are responsible for their hygroscopic nature. To counteract this, surface modification is required to improve the stability of aerogels. This* is normally performed using silane containing hydrophobic organic groups (TMCS, HMDZ, HMDSO, TMMS, DMDMS, MTMS, etc.), which confers a hydrophobic nature to aerogels [13,14,15]. The structures of different silylating agents are represented in Scheme 2. These precursors react with Si–OH on the surface of the wet gel, forming methylsilyl groups. Recently, Rao has reviewed synthesis and applications of silica-aerogel-based silica aerogels [16].

Different methods to prepare hydrophobic silica aerogels were intensively studied by many authors. It was observed that TMCS drastically reduced the shrinkage of aerogels derived from sodium silicate, yielding a sturdy light-weight material with high porosity [17,18]. A crack-free silica aerogel was prepared using subcritical drying via surface modification of wet gels using IPA/TMCS/*n*-hexane solution [19]. The obtained aerogels possessed high porosity and low density (0.12–0.15 g/cm^3^). More recently, Park and coworkers studied the effect of silylation by varying the molar ratios of silylating agents (MTMS, DMDMS, and TMMS) [20] and further explained the silylation mechanism based on the molecular structures by considering the number of methoxy/methyl groups. They concluded that MTMS was not helpful for surface modification, whereas DMDMS showed a similar degree of silylating ability as TMCS. Aerogels modified with DMDMS exhibited high porosity (90%), high surface area (475 m^2^/g), low bulk density (0.19 g/cm^3^), and high hydrophobicity with a water contact angle (WCA) of 132°.

Parvathy Rao and coworkers systematically optimized conditions for synthesizing aerogels from sodium silicate by varying acid catalyst and silylating agents [21,22]. The physical properties of these aerogels were affected by the strength and concentration of acid [23]. Strong acids (HCl, H_2_SO_4_) resulted in higher shrinkage (70–95%) and required longer gelation time. In contrast, weak acids (citric acid, tartaric acid) resulted in low shrinkage (34–50%) due to the systematic network formation during gelation. Additionally, the percentage of silylating agent in the mixture, time interval of addition, and volume of silylating mixture affected the density, porosity, and optical transparency of the aerogels [24]. The mixture of TMCS and HMDSO provided transparent, hydrophobic (WCA 152°) aerogels with low density and low refractive index. In parallel, Bhagat and coworkers reduced the processing time (1 day) by utilizing the co-precursor method for surface modification in the hydrogel to obtain aerogel beads [25]. The beads were obtained with pore diameters ranging from 3.2 to 4.9 nm and a specific surface area of 591 m^2^/g. A simple, cost-effective method was introduced to obtain silica aerogels via surface modification of hydrogels with a very low concentration of HMDZ [26,27]. Initially, sodium silicate was treated with the mixture of HNO_3_/HMDZ, where HNO_3_ promoted the hydrolysis of HMDZ. Both surface modification and solvent exchange occur simultaneously and, as a result, superhydrophobic aerogels were obtained within 5 h. Table 1 summarizes the properties of silica aerogels prepared from various precursors.

In another report, transparent low-density silica aerogel beads were fabricated through acid-base sol-gel polymerization of sodium silicate via ball dropping method [28]. The surface area and the pore volume of the aerogel beads increased with an increase in the volumetric percent of TMCS, and with 10%V TMCS aerogel beads with low density (0.081 g/cm^3^) and high surface area (917 m^2^/g) were obtained. Recently, Park et al. synthesized silica aerogels to improve the optical transmission using a two-step sol-gel process via ambient pressure drying [29]. They observed that the aerogels prepared using 3 wt% of silica had low density (0.11 g/cm^3^), low thermal conductivity (0.12 W/mK), and a surface area of 590 m^2^/g. The preparation of aerogels with sodium silicate via APD is the cheapest method. However, surface modification and solvent exchange steps make this process tedious. In addition, there has not been a report on monolithic aerogels from sodium silicate via APD due to considerable crack formation. The application of monolithic silica aerogels would be enhanced by future developments in the chemical and engineering processes for solving these issues.

#### 1.1.2. Tetraalkoxysilane-Based Aerogels

The process for aerogel production using the Kistler method is tedious and time consuming. The whole process of aerogel preparation from sodium silicate takes more than a week due to troublesome washing and solvent exchanging steps. There was no follow-up interest in the field of aerogels until the 1960s. Interestingly, in 1968, a research team headed by Teichner improved the process of making aerogels by dissolving tetramethoxysilane (TMOS) in methanol and was able to prepare aerogels within 12 h [30]. This method eliminated the formation of inorganic salt and the need for a solvent exchange step. Following this, multiple research groups began working on silica aerogels for a number of different technological applications.

**Figure 3:**
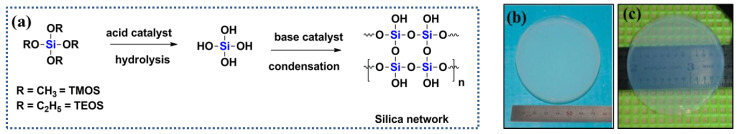
(**a**) Synthesis of silica aerogels from tetraalkoxysilane, (**b**,**c**) translucent and transparent monolithic aerogels derived from TMOS. Reprinted with permission from Refs. [33,34]. Copyright 2007, 2016 Elsevier.

The chemical reactions of an alkoxysilane precursor during sol-gel polymerization are described in Figure 3a. The hydrolysis reaction replaces the alkoxy group with hydroxy groups, and subsequently, the silanol groups form siloxane bonds (Si-O-Si) via condensation reactions along with the release of alcohol or water as byproducts. In most cases, condensation initiates in parallel with the hydrolysis reactions and persists during the whole sol-gel process. Additional linkage of silica tetrahedral species via polycondensation reactions leads to open and cyclic oligomers, which subsequently forms a network of silica gel. Intermediate species with functionalities S-OR (R is typically methyl or ethyl) and Si–OH remain in the final gel structure. Due to the relatively low reactivity of alkoxysilanes compared with other metal alkoxides, hydrolysis proceeds rapidly in the presence of acid or base catalyst. Generally, mineral acids or ammonia are used in the sol-gel reaction. Gel formation from alkoxysilanes can proceed using a one-step process in the presence of acid or base or using a two-step process in which hydrolysis and polycondensation are catalyzed separately by acid and/or base. More extended controls over the microstructure and pore size of aerogels are possible with the two-step acid–base reaction, since the kinetics and equilibrium in hydrolysis and polycondensation can be controlled separately.

Hrubesh et al. proposed a two-step process of TMOS and obtained transparent silica aerogels with a wide density range [31]. To reduce processing time and enhance the sol-gel reactions of TMOS in supercritical CO_2_ drying method, Loy and coworkers fabricated aerogels using formic acid as an alternative to water [32]. Large monolithic hydrophobic silica aerogels were easily prepared from TMOS by controlling the molar ratio of catalysts [33,34]. Low-density translucent and transparent monoliths were easily obtained from TMOS (Figure 3). However, the toxic nature of TMOS is a barrier for its usage for industrial productions.

Concurrently, many authors studied formulation with tetraethoxysilane (TEOS), which is mild, less harmful, and a convenient precursor for the synthesis of silica aerogels [35,36,37,38]. Rao’s research team studied the effect of various parameters (such as catalyst concentration and pH) and prepared transparent aerogels using TEOS as the precursor [39,40]. The physical properties of aerogels derived from TEOS, TMOS, and Na_2_SiO_3_ were compared [41]. They concluded that the TMOS-derived aerogel possessed excellent hydrophobicity with the highest water contact angle (149°). Later, Carroll et al. prepared silica aerogel monoliths using rapid supercritical extraction, adapting a TEOS-based recipe from Rao et al. [42]. Light-weight aerogel materials were prepared by varying the aging period under ambient pressure, which increased the stiffness [43]. Rao and coworkers further studied the effect of different solvents in the solvent exchange steps [44]. Materials possessing low density, high porosity (96.5%), and low thermal conductivity (0.090 W/mK) with good optical transmission were successfully obtained from TEOS by employing HMDZ as a silylating agent. They obtained superhydrophobic aerogels (WCA 172°) when xylene was used for solvent exchange; however, the level of hydrophobicity decreased over time. Lu and coworkers developed TEOS-derived monolithic silica aerogels using ambient pressure drying through a multiple modification approach using TMCS [45]. They concluded that the multiple treatments of the wet gel helped to reduce drying-induced surface tension force to maintain integrity and high porosity.

In 1995, Prakash and coworkers developed an ambient pressure drying protocol to prepare aerogel films using TEOS as precursor followed by surface modification with TMCS [46,47]. They obtained aerogel films with 98.5% porosity. This method enables the spring-back phenomenon, in which temporarily shrunk gel networks recover to their original form like a sponge. Later, Kim et al. fabricated TEOS-derived aerogels modified with TMCS using isopropanol as preparative solvent via APD. The resulting materials possessed low densities (0.041 g/cm^3^) with high surface areas (1150 m^2^/g) [48]. However, the conventional APD alcogels need tedious repetitive gel washing and solvent exchange steps. To reduce the processing time, Mahadik et al. developed TMCS-modified aerogel granules derived from TEOS by using various base catalysts (TBAF, TEAF, TMAF, NH_4_OH, and NH_4_F) to obtain aerogel [49]. The combination of NH_4_OH and NH_4_F catalysts resulted in transparent, low density (0.067 g/cm^3^), hydrophobic aerogels with high optical transmittance (90%). Furthermore, they fabricated TEOS-based silica aerogels via a two-step sol-gel process and demonstrated that the surface free energy of aerogels can be tuned by modifying their surface using varied concentrations of TMCS and HMDZ silylating reagents over a wide range (5.5892 to 0.3073 mJ/m^2^) [50]. They observed an increase in WCA (123° to 155°) with a corresponding reduction in surface energy. Recently, Cok and Gizli prepared TEOS-derived silica aerogels via two-step surface modification using different silylating agents (TMCS, MTMS, MTES, and MEMO) followed by APD [51]. Among different silanes, MTES showed a homogenous pore network, high surface area (964 m^2^/g) and high hydrophobicity (WCA 137°).

#### 1.1.3. Trialkoxysilane-Based Aerogels

Another interesting precursor for polysiloxane aerogels is trialkoxysilane, which empirically leads to hydridosilsesquioxane (HSQ). Since a hydrogen atom is not classified as an organic substituent, HSQ is included in this section. The hydrogen substituent is small and possibly forms hydrogen bonds with silanols. The sol-gel system of HSQ consequently shows similar behaviors to the TMOS-based one. The Si-H is vulnerable to hydrolysis during the sol-gel process. Under basic conditions, nucleophilic attack of hydroxide on the trialkoxysilane precursor results in cleavage of the Si-H moiety. This restricts the sol-gel route for HSQ to neutral or weakly acidic paths. Ozin and coworkers made considerable progress in the preparation of periodically mesoporous particles from triethoxysilane (HTES) in an acid-catalyzed system with Pluronic P123 [(poly(ethylene oxide)-*block*-poly(propylene oxide)-*block*-poly(ethylene oxide), denoted as EO_20_PO_20_EO_20_] as surfactant [52]. The resulting SiO_2_ nanocomposite materials were brightly luminescent and exhibited size-controlled photoluminescence, which bodes well for the development of light emitting devices and biological sensors. Later, in 2013, Kanamori and coworkers fabricated HSQ monoliths with well-defined macropores and mesopores from trimethoxysilane (HTMS) in the presence of poly(ethylene oxide) as phase separation inducer [53]. These HSQ materials offer surface reactivity such as reduction, which is advantageous for preparing metal nanoparticle-supported porous materials, and were shown to be promising as heterogeneous catalysts, exhibiting reusability and recyclability.

Despite their outstanding properties and potential applicability, most of the conventional silica aerogels suffer from low mechanical strength and their hydrophilic nature makes them unstable in atmospheric conditions, which makes processing and handling difficult [54]. From the viewpoint of practical applications, monolithic materials are easier to be handled compared with powder forms. Alkoxysilanes (TEOS or TMOS) are the most favored precursors as monoliths are easily obtained. However, the alkoxysilane precursors are significantly more expensive compared with sodium silicate. The combination of sodium silicate and ambient pressure drying is the most promising route to produce silica aerogels at low cost. At present, silica aerogel granules are manufactured by Cabot Aerogels on an industrial scale using sodium silicate [55].

**Table 1:** Some examples of precursors used for synthesis of silica aerogels.

Precursor/ Silylating Agents	Drying	Properties	Ref.
Na_2_SiO_3_	SCF	Density of 0.1 g/cm^3^, 95% porosity, hard and brittle.	[56]
Na_2_SiO_3_-TMCS	APD	Density of 0.066 g/cm^3^, 95% porosity, hydrophobic (145°).	[17,21]
Na_2_SiO_3_-DMDMS	APD	Density of 0.19 g/cm^3^, porosity (90%), SSA of 475 m^2^/g with contact angle 132°.	[20]
Na_2_SiO_3_/TMCS/HMDSO/HMDZ	APD	Transparent, density of 0.042–0.18 g/cm^3^, 90–97% porosity, thermal conductivity of 0.05–0.192 W/mK, SSA of 450–590 m^2^/g.	[22]
Na_2_SiO_3_-TMCS	APD	Low thermal conductivity of 0.09 W/mK, density of 0.086 g/cm^3^, 95% porosity, hydrophobic with contact angle 148°.	[23]
Na_2_SiO_3_-HMDZ	APD	Aerogel powder with density of 0.1–0.3 g/cm^3^ and SSA 559–618 m^2^/g.	[26]
TMOS	SCF	Density of 0.039–0.2 g/cm^3^ and SSA of 123–947 m^2^/g.	[33,34]
TEOS	SCF	Hydrophobic (145°), density of 0.12–0.18 g/cm^3^, SSA 620 m^2^/g.	[40]
TEOS-HMDZ	APD	Superhydrophobic (160°) with thermal conductivity of 0.07 W/mK.	[44]
TEOS-TMCS	APD	Monoliths, with high porosity (97%), SSA of 777 m^2^/g, thermal conductivity of 0.036 W/mK, hydrophobic with contact angle 143°.	[45]
TEOS-TMCS	APD	Aerogel films with high porosity (98.5%).	[47]
TEOS-TMCS	APD	Density of 0.074–0.041 g/cm^3^ and surface area 1150 m^2^/g.	[48,50]
HTES	APD	Improved mechanical properties with surface area of 434 m^2^/g.	[52]
HTMS	APD	Monolithic aerogel with high surface area ~800 m^2^/g.	[53]

### 1.2. Organically Modified Silica Aerogels

Hybridization is a promising way to improve the mechanical properties of silica aerogels to extend their applications. Nevertheless, synthesis of organic–inorganic hybrids from organotrialkoxysilane is challenging due to the hydrophobicity of condensates and steric effects exerted by the organic moiety. Therefore, a significant fraction of recent studies have been associated with the development of hybrid aerogels [57,58,59,60]. These hybrid materials are categorized into two classes, class I and class II, depending on the connectivity between organic and inorganic counterparts [60,61]. Class I hybrid aerogels are prepared by mixing two separate organic and inorganic compounds via sol-gel process. These composites are the results of physical interactions such as van der Waals forces, electrostatic forces, and hydrogen bonding between organic and inorganic phases [62].

The class II hybrid materials involve strong chemical interactions (such as covalent and iono-covalent bond) between organic and inorganic phases. There are two types of hybridization strategies: (1) employment of organoalkoxysilanes as a precursor, and (2) formation of composites with polymers [63] or structural supports. Silica aerogels can be strengthened by introducing organic groups using a co-precursor into an inorganic framework through a Si-C bond [64]. The resulting hybrid materials are termed “Organically Modified Silica” (ORMOSILs) or organically modified ceramics. The organic group, which comprises around 40–60% of the material, remains an integral part of the network. They can be varied in terms of the length, rigidity, and geometry of the substituent, thus providing an opportunity to modulate the bulk properties of aerogels. Typical examples of ORMOSILs include silsesquioxanes and bridged silsesquioxanes, depending on the precursor used. The synergetic combination of organic and inorganic moieties in a single-phase material provides unique possibilities to tailor the thermal, mechanical, and optical properties. The presence of nonpolar alkyl or aryl groups attached to a silica-based network may result in flexible three-dimensional networks. The general preparation method, properties, and applications of organically modified aerogels can be found in some outstanding review papers [65,66].

#### 1.2.1. Silsesquioxanes

**Figure 4:**
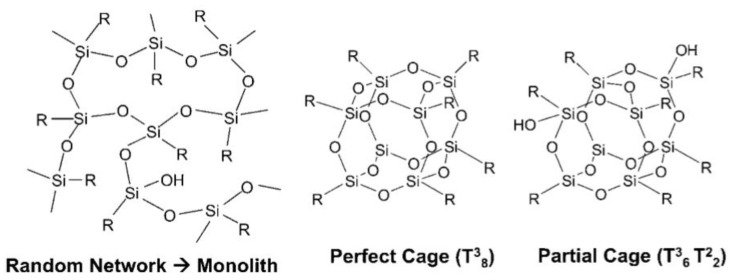
*Typical structure of silsesquioxanes derived from trialkoxysilanes*.

**Scheme 3:**
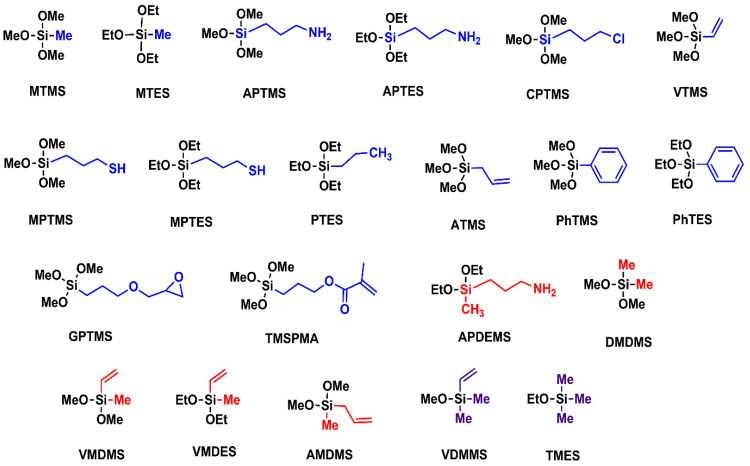
Organosilane precursors used in the preparation of silsesquioxanes.

Among the family of hybrid aerogels, silsesquioxane are based on compounds with RSiO_1_._5_ repeat units, and this group has grown dramatically [67,68,69]. The first commercialization of silicones began with silsesquioxane chemistry. Silsesquioxanes derived from trifunctional silanes structurally exhibit siloxane networks or cages with varied pendent groups (Figure 4). Typically, there are two types of materials: polymers based on random networks (T resins) and oligomeric molecules known as polyhedral oligosilsesquioxane (POSS). The structures of monofunctional (R_3_SiX, X = alkoxy, halogen, etc), difunctional (R_2_SiX_2_), and trifunctional (RSiX_3_) organosilane used in the preparation of silsesquioxane are described in Scheme 3. All these precursors are characterized by the presence of Si–O covalent bonds. The network material can be formed from RSiX_3_ via condensation with tetrafunctional monomers such as TEOS or TMOS. The resulting morphology of hybrid material is modulated via RSiX_3_/TEOS ratio. Due to high thermal and mechanical stabilities with variable porosity, silsesquioxanes are used in various applications such as ionic liquids [70], organic semiconductors for electronics [71], electrolytes for lithium ion batteries [72], water desalination [73], gas/liquid phase separation [74], and optical materials [75].

In an altogether different approach to develop low-density aerogels, Novak et al. introduced the concept of interpenetrating networks of inorganic and organic moieties. Polyvinylpyridine (PVP) was introduced into silica networks through the addition of CuCl_2_, and the organic polymer was generated in situ via radical polymerization of a vinyl monomer [76]. Later, Kramer et al. successfully reinforced silica aerogels with a silicone using TEOS with varying amounts of polydimethylsiloxane (PDMS) via a two-step acid/base catalyzed process [77]. These organically modified aerogels displayed optical transparency, improved mechanical strength, and possessed a surface area of up to 1200 m^2^/g.

The gelation behavior of trialkoxysilane is much more complicated than that of tetraalkoxysilanes due to cyclization and premature phase separation [78]. In most cases, using only trialkoxysilane as a precursor results in failure to form a monolithic gel because of the steric hindrance and hydrophobicity derived from the organic moiety [79]. *Schubert and Hüsing fabricated a series of hybrid aerogels using tetraalkoxysilane with a broad range of trialkoxysilanes including MTMS, MPTMS, APTMS, GPTMS, and MAPTMS [80,81,82,83]. They observed higher shrinkage and longer gelation time with an increasing fraction of trialkoxysilanes due to the incomplete hydrolysis and condensation reaction of the trialkoxysilanes. Moreover, in most cases, aerogels became turbid with the increasing concentration of trifunctional monomers due to cyclization and macroscopic phase separation in polar solvents [79,84], which prevented the formation of 3D random networks and resulted in the formation of domains larger than submicrons, which lowered the visible-light transmittance.*

**Figure 5:**
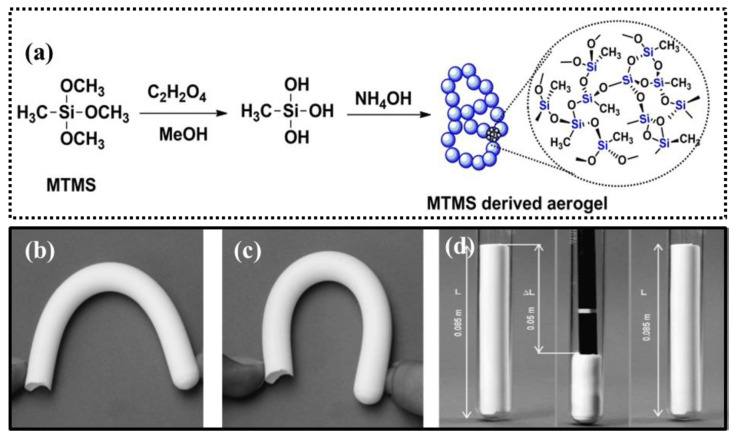
(**a**) Synthesis of MTMS-based flexible silica aerogel. Aerogels prepared at two different molar ratios MeOH/MTMS (**b**) 28 and (**c**) 35. (**d**) Photograph showing three states of flexible aerogel sample. Reprinted with permission from Ref. [90], copyright 2006 Elsevier.

The fabrication of flexible superhydrophobic aerogels has attracted extensive interest from a practical point of view. In 2006, Rao et al. reported a novel synthetic approach to prepare flexible aerogels using an MTMS precursor *(*Figure 5). The nonpolar methyl groups present in MTMS impart hydrophobicity, and the reducing number of Si-O-Si bonds leads to fewer cross-linked structures, which provides mechanical flexibility to the aerogels as shown in Figure 5. They used a *mixture of trialkoxy and tetraalkoxysilanes to develop transparent, superhydrophobic aerogels [85,86,87,88]. Generally, MTMS-derived aerogels are prepared via a one-step base catalyzed or two-step acid-base catalyzed sol-gel process. It was observed that the two-step process is more suitable to obtain a monolithic polysiloxane network*. However, increasing the molar ratio of MTMS/TMOS decreased the transparency and *specific surface area with a Young’s modulus of 0.03–0.06 MPa* due to *enhanced* phase separation. In later studies, Bhagat et al. produced monolithic MTMS-derived aerogels via ambient pressure drying, but they did not provide a detailed discussion on the flexibility performance [89].

*When employing MTMS as a single precursor,* the concentration of MTMS influences the mechanical properties of the aerogels, and Young’s modulus decreased from *0.141* to *0.0343 MPa [90]. As the* molar ratio *of* MeOH/MTMS *increased from 14 to 35, there was an increase in the flexibility and a decrease in the density. This occurred because the silica networks were separated from each other and linear networking was enhanced*. These random networks derived from MTMS *are termed as polymethylsilsesquioxane (PMSQ). They are composed of polymeric random networks with the ideal chemical formula CH_3_SiO_1_._5_. Although both silica and PMSQ aerogels consist of Si-O-Si bonds in their networks, their properties differ in mechanical durability due to the incorporated methyl groups.* Recently, Borzecka et al. investigated the kinetics of formation of MTMS-based silica aerogels prepared using sol-gel polymerization and described the dynamics of the condensation reaction [91]. They concluded that both a numerical model and experimental test can be used in the mass prediction of aerogels during modification of the materials.

*Recently, Yun and coworkers* prepared large-sized (240 cm^3^) monolithic MTMS-based aerogels via facile sol-gel method [92]. *The resulting APD dried* superhydrophobic *aerogels showed* macropore structures with low density, low thermal conductivity (0.036 W/mK), and good thermal stability. It was observed that the Young’s modulus of the aerogels increased (0.043 to 1.102 MPa) with an obvious increase in the density (0.075 to 0.14 g/cm^3^). The simple fabrication method and the superior performance of these aerogels *make them* useful in long-term and large-scale thermal applications. *Hydrophobic aerogels based on TEOS are prepared by incorporating ETES and PhTES as co-precursors with varied molar ratios [93,94]. Depending on the molar ratio (<0.1), transparent monolithic aerogels were obtained.*

In pursuit of aerogels with improved mechanical properties, Roig et al. integrated organically modified silica aerogel by using TMOS/MTMS and TMOS/TMSPMA [95]. They found that surface area increased with an increase in the concentration of MTMS and the contact angle reached 160° in the TMOS/MTMS system. Meanwhile, TMSPMA-based aerogels did not withstand high temperature supercritical drying. Macroporous PMSQ monolithic materials with various sol-gel systems (acid/base and acid/acid method) containing MTMS were fabricated by Dong et al., leading to improvements in terms of controlling phase separation and gelation time [96,97,98]. They were able to produce bimodal and trimodal PMSQ monoliths, depending on the conditions employed.

The practical applications of aerogels are limited due to the reduced transparency and the lack of control over thickness and porosity. Incorporating trifunctional monomers with alkyl trialkoxysilanes makes the resultant aerogel surface hydrophobic, but sacrifices the transparency and surface area due to induced macroscopic phase separation. In 2007, Kanamori et al. explored phase separation-gelation behavior of MTMS-based aerogels using various ionic (CTAC, CTAB) and nonionic (Pluronic F127) surfactants and obtained transparent PMSQ aerogels with uniform porosity for the first time [99,100,101,102]. The molecular structures of surfactant are presented in Table 2**.** Cationic surfactant CTAB interacts with the silica domain by settling the polar head groups toward the silica, which effectively suppresses the macroscopic phase separation, while nonionic surfactants facilitate hydrogen bonding between the silanol groups (Si–OH), and urea helps to accelerate the polymerization of MTMS by raising the solution pH. Because of the elastic nature of the PMSQ network, the PMSQ aerogels obtained displayed reversible “spring-back” behavior against compressive deformation. They also exhibited high porosities (94%) with low bulk densities (0.1 g/cm^3^). Later, Kanamori and coworkers explored the effects of the molecular structure of nonionic surfactants on the properties of the resultant PMSQ aerogels by employing surfactants of different molecular weights [103]. They concluded that transparent aerogels cannot be obtained from surfactants with very low molecular weight (L35) or very high hydrophobicity (P123).

Xiaodong and coworkers produced flexible aerogels by applying MTMS/TEOS co-precursors and CTAC as surfactant via ambient pressure drying technique [104]. The resulting aerogels showed excellent flexibility, and exhibited a hydrophobic nature (CA of 153.9°) with a superior thermal insulating property. More recently, Li and coworkers developed MTMS-based monolithic silica aerogels in the presence of CTAB as surfactant in pure water within 4 h [105]. They also investigated the effect of MTMS/H_2_O ratio, CTAB content, and NH_3_·H_2_O concentration on the properties of aerogels. They observed that aerogels with higher volume ratios of H_2_O/MTMS exhibit larger Young’s modulus and smaller compressive stress because of the difference in microstructure. This process could help in the fast massive production of aerogels.

**Scheme 4:**
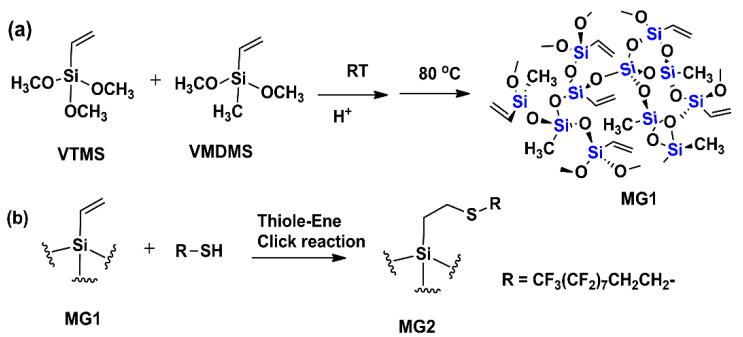
(**a**) A synthetic scheme for vinyl-modified gels (MG1) from VTMS and VMDMS. (**b**) Surface modification to add perfluoroalkyl groups (MG2). Reproduced with permission from Ref. [110]. Copyright 2013 Wiley.

There are numerous reports on aerogels with trifunctional alkoxysilane, but very few reports on mono- and dialkoxysilanes because of their tendency toward phase separation at levels that are too high, arising from the hydrophobicity of the network. To obtain low-density bendable materials, Hayase et al. developed “marshmallow-like” aerogels from an MTMS-dimethyldimethoxysilane (DMDMS) co-precursor system using CTAC surfactant [106,107,108]. As DMDMS concentration increased, samples recovered their original shape after unloading, when compressed to 80% of their original size. These marshmallow-like gels showed bending flexibility and can be used in oil–water separation. Variations of the marshmallow-like gels have been demonstrated by using VTMS, MPTMS, and PhTMS, among others. The marshmallow-like gel can also be tailored in a powder form [109]. A more advanced surface design can be achieved when precursors with reactive groups are employed to prepare flexible aerogels. The co-condensation reaction between VTMS and VMDMS followed by the thiol-ene reaction on the surface is illustrated in Scheme 4. Perfluoroalkyl groups were introduced for surface modification [110]. These flexible material designs are beneficial for developing multifunctional porous materials. Table 3 summarizes the physical properties of organically modified aerogels.

The use of surfactants suppresses the phase separation and, as a result, flexible, superhydrophobic, and transparent aerogels were obtained, but in general surfactants are very expensive and the residual surfactant causes serious shrinkage and cracks in the monoliths during drying. Therefore, Kanamori et al. developed a surfactant-free method to obtain hydrophobic PMSQ aerogels by copolymerizing with N-[3-(trimethoxysilyl)propyl]-N,N,N-trimethylammonium chloride (TMAC), which helps in suppressing undesirable phase separation [111]. The obtained materials revealed low-density, high visible-light transmittance, and good thermal insulation (0.0136 W/mK) properties equivalent to those prepared in the presence of surfactant.

To ascertain the effect of the molar concentration of precursor and drying parameters on the properties of aerogels, Durães et al. fabricated aerogels by varying the molar ratio of MTMS, MTES, and ETMS [112,113,114]. The ETMS co-precursor led to a significant increase in the product density with a reduction in surface area. Therefore, MTMS is a more suitable precursor, yielding hydrophobic aerogels with low density, average surface area (400 m^2^/g), and good flexibility. Aerogels prepared from MTMS as a precursor followed by ambient pressure drying not only maintain excellent monolithic properties, but also reduce the manufacturing cost [115]. However, the methyl group does not play an effective role in improving the compressive strength of silica aerogel. Yang et al. fabricated silica aerogel using MTMS and VTES as precursor and demonstrated that the replacement of methyl with vinyl groups enhances the mechanical properties (compressive stress 0.57 MPa) [116]. In addition, including propyl groups in the underlying silica skeleton of MTMS-derived aerogels can also improve their flexibility such that they can be compressed to 70% of their original height [117]. Recently, Smitha et al. fabricated hydrophobic aerogel coating made from a composite of MTMS and GPTMS (1:0.5) and demonstrated oil adsorption properties [118]. The authors also prepared low density porous silica aerogel by varying the molar ratio of TEOS/GPTMS through ambient pressure drying [119]. Recently, *Hüsing and coworkers prepared flexible aerogels with reactive functional groups by co-condensing MTMS with various organosilanes (VTMS, CPTMs, MPTMS, TMSPMA) with the aid of CTAB surfactant [120]. The ratio of MTMS and organosilane was kept above 8.5:1.5 to retain their interesting properties. The resulting functional gels exhibited excellent elastic compression behavior up to 60%. They observed that density, porosity, and linear shrinkage remained the same with the increasing the size of functional groups, whereas surface area decreased with further modification of gels.*

Silica aerogels with more flexibility have been fabricated using methyltriethoxysilane (MTES) as a sole precursor. Nadargi and coworkers prepared monolithic aerogels by employing a two-stage acid-base catalyzed sol-gel process followed by supercritical drying. Aerogels with different densities were obtained by varying the molar ratio of MeOH/MTES(S) [121,122]. It was observed that low dilution of MTES led to less flexibility, and the aerogels with the highest S ratio showed the highest flexibility. However, further bending of these samples resulted in crack formation. Furthermore, Aravind et al. synthesized porous hydrophobic MTES-based silica aerogel under ambient pressure drying with a surface area of 727 m^2^/g [123]. However, the Young’s modulus and shrinkage were not mentioned. Cui and coworker studied the temperature-dependent microstructure of silica aerogels using 0.5 molar ratios of MTES/TEOS. Aerogels were subject to heat at different temperatures (200–500 °C for 2 h). They found temperature-driven transition from hydrophobic to partially hydrophilic to completely hydrophilic [124]. Transparent flexible silica aerogels are prepared by replacing the traditional solvent (alcohol) with water, using MTES and CTAB, via an acid-base two-step method [125]. The resulting flexible, transparent, hydrophobic (151°) silica aerogels exhibited low thermal conductivity (0.0215 W/mK) with an initial decomposition temperature of up to 511 °C.

**Figure 6:**
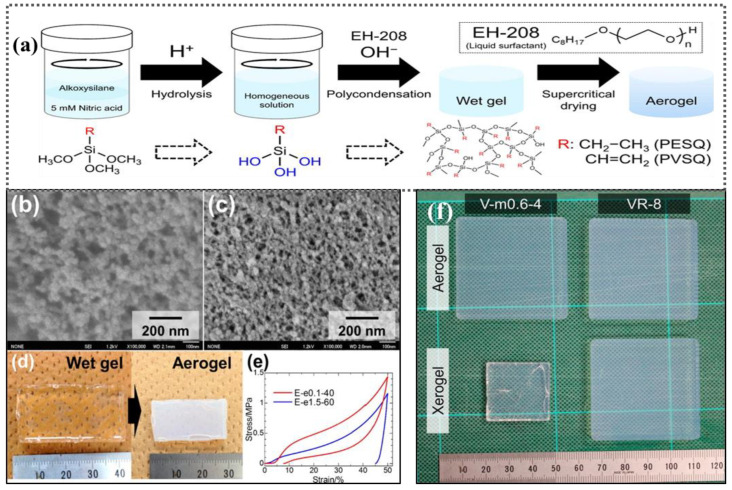
(**a**) Synthesis of PESQ and PVSQ aerogels, (**b**,**c**) SEM images of PESQ aerogels, (**d**) PESQ aerogel showing large volume shrinkage after SCF drying, (**e**) stress–strain curves on uniaxial compression of PESQ aerogels at different concentrations, (**f**) photograph of PVSQ aerogels and xerogels. Reproduced with permission from Ref. [127]. Copyright 2016 American Chemical Society.

Alternatively, organotrialkoxysilanes with longer alkyl groups (ethyl, vinyl, and propyl) are used to prepare aerogels. Itagaki et al. have investigated the phase separation behavior by varying the molar ratio of VTMS and TMOS as co-precursors in the presence of formamide under an acidic condition and prepared monolithic amorphous alkylsiloxane gels with well-defined macropores [126]. Shimizu et al. developed transparent hydrophobic polyethylsilsesquioxane (PESQ) and polyvinylsilsesquioxane (PVSQ) aerogel using ETMS and VTMS precursors in the presence of nonionic surfactant EH-208 (polyoxyethylene 2-ethylhexyl ether) [127], as represented in Figure 6. Micrographs clearly show a thick gel skeleton with large pores (100 nm), and the presence of a coarsened porous structure causes visible-light scattering. Typical stress–strain curves of PESQ aerogels are presented in Figure 6e, which exhibits flexible behavior on uniaxial compression of up to 50% without collapse. The mechanical properties of PVSQ aerogels are further strengthened by radical polymerization of vinyl groups in the solid network using AIBN radical initiator. These aerogels had the desired transparency and compressibility. However, the density was high and they possessed low elastic modulus.

Trifunctional precursors with long alkyl chains are also employed to prepare silica aerogels. Yang et al. fabricated superhydrophobic silica aerogel by incorporating PTES/TEOS co-precursors employing ambient pressure drying [128]. The resulting aerogels exhibited low density, high surface area, and can endure up to 70% maximum linear compression with few cracks. They also possess a high absorption capacity (8–10 times their own weight). Recently, Li and coworkers prepared *silica-polymethylmethacrylate* composite *aerogel using TMOS and TMSPMA* via load transfer across a static electric phase interface [62]. Rao et al. used monofunctional silica precursor trimethylethoxysilane (TMES) with TMOS to prepare hydrophobic silica aerogels [129]. TMES/TMOS molar ratio (S) was varied from 0 to 2.35. As the S value increased, the hydrophobicity of the aerogels increased, but the optical transmission diminished from 93% to less than 5% in the visible range.

**Table 2:** Physical properties of ionic and nonionic surfactants.

Type	Surfactant	Molecular Structure	Appearance	Ref.
Cationic	CTAC	*CH_3_(CH_2_)_15_N(Cl)(CH_3_)_3_*	transparent	[99]
	CTAB	*CH_3_(CH_2_)_15_N(Br)(CH_3_)_3_*	transparent	[99]
Nonionic	P123	EO_20_PO_70_EO_20_	opaque	[103]
	P105	EO_37_PO_56_EO_37_	transparent	[103]
	L35	EO_11_PO_16_EO_11_	opaque	[103]
	F127	EO_106_PO_70_EO_106_	transparent	[99]
	F108	EO_132_PO_50_EO_132_	transparent	[103]
	F68	EO_78_PO_30_EO_78_	transparent	[103]
	EH-208	C_8_H_17_O-(C_2_H_4_O)_n_-H	transparent	[127]

**Figure 7:**
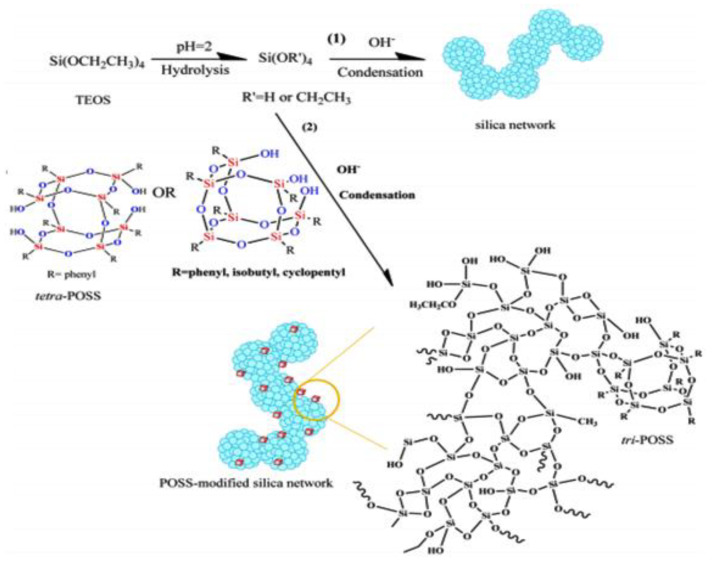
Formation of unmodified silica networks (path 1) and modification of silica networks by POSS molecules (path 2). Reprinted with permission from Ref. [133]. Copyright 2012 American Chemical Society.

**Figure 8:**
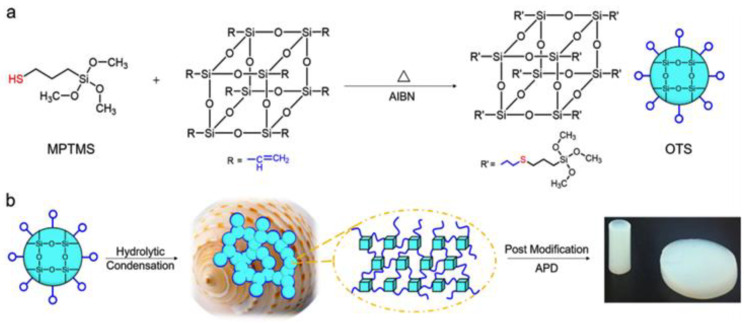
(**a**) Synthesis of precursor and (**b**) final hybrid aerogel. Reprinted with permission from Ref. [134]. Copyright 2019 American Chemical Society.

Polyhedral oligomeric silsesquioxane (POSS) is also used to construct novel organic–inorganic materials. Many research groups have developed unique silica aerogels based on POSS which have resulted in some inspiring improvements to aerogel properties [130,131,132]. Jana et al. evaluated a POSS molecule carrying phenyl, iso-butyl, and cyclohexyl organic side groups as a multifunctional reinforcing agent within the silica aerogel [133]. Schematic illustration of TEOS and POSS molecules are displayed in Figure 7. The compressive modulus increased six-fold with less than 5 wt% trisilanol phenyl-POSS, with negligible increases in density. Recently, Li and coworkers prepared octa [2-((3-(trimethoxysilyl)- propyl)thio)ethyl]silsesquioxane (OTS)-based superhydrophobic aerogels using MPTMS and POSS via thiol-ene click chemistry and studied the effects of OTS:H_2_O molar ratio on the physical properties of aerogels (Figure 8) [134]. Benefiting from the alternating rigid inorganic nanocage skeleton, the resulting flexible OTS aerogels had high surface area (542–834 m^2^/g) and high compression strengths (4.96–6.48 MPa), with a better compression modulus (18.79–25.84 MPa). The good oil–water separation efficiency of OTS aerogels can be utilized for practical application.

**Figure 9:**
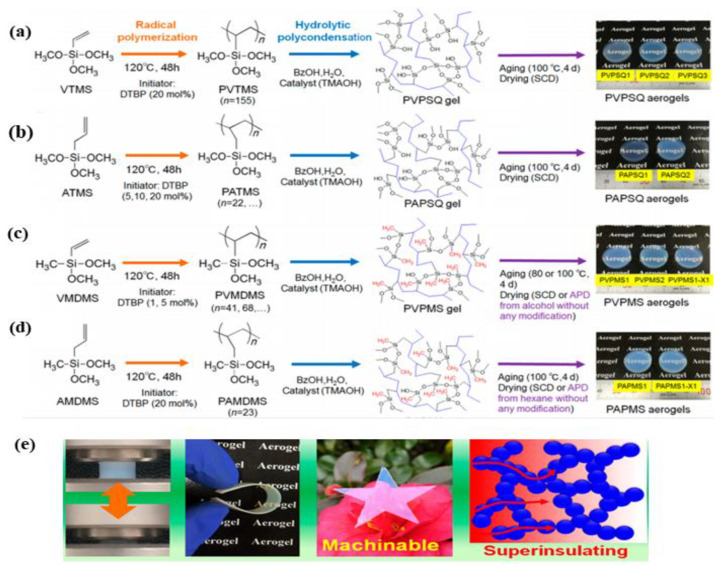
Double-cross-linking method and resultant aerogels. (**a**) PVPSQ, (**b**) PAPSQ, (**c**) PVPMS, and (**d**) PAPMS from different precursors, (**e**) bending flexibility and excellent machinability of PVPMS aerogels. Reproduced with permission from Ref. [137]. Copyright 2018 American Chemical Society.

Silica aerogels are made using monomeric or polymeric precursors through bimodal or spinodal decomposition phase separation. Kanamori et al. prepared a series of materials using pre-polymerized precursor before gelation [135]. They concluded that using pre-polymerized precursor significantly improves the thermal and mechanical properties of resulting aerogels. As a follow-up to this, a new class of transparent, superflexible silica-based aerogels has been prepared using pre-polymerized silica precursor prior to gelation. This novel approach was first introduced by Zu et al. They initially polymerized monofunctional (VDMMS), bifunctional (VMDMS, VMDES, and AMDMS), and trifunctional (VTMS, ATMS) precursors and later cross-linked the network with siloxane bonds to form a polymeric backbone in the presence of a di-tert-butyl peroxide (DTBP) initiator via ambient pressure drying (Figure 9) [136,137,138]. The resulting aerogel network consisted of polysiloxane as cross-linker and hydrocarbon chain as the backbone, which led to excellent flexibility and processability while maintaining the superinsulating properties (15.2 mW/mK). This double-cross-linking approach significantly enhanced the skeletal structure of aerogels to resist cracking during the drying process. This method is low-cost and is scalable from molecular level to robust networks, and the aerogels exhibit hydrophobicity [139]. However, this strategy requires higher temperatures and a prolonged aging process.

Polyvinylpolymethylsiloxane (PVPMSA) aerogels were reported by Feng et al. using a DTBP initiator and following Zu’s double-cross-linking approach, and they systematically studied the effect of temperature and pressure on the thermal properties [140]. They concluded that the thermal conductivity of PVPMSA aerogels is greatly affected by the temperature. Polyvinylmethyldimethoxysilane (PVMDMS)-reinforced MTMS aerogels were also developed, which showed elastic recovery properties and super high surface area (1479 m^2^/g), mainly due to the long aliphatic hydrocarbon chain and the presence of excess methyl groups [141]. Furthermore, Park et al. used pre-polymerized VTMS and GPTMS precursors to create a nonparticulate structure through spinodal decomposition phase separation [142,143]. This approach reduced the aging process and offered aerogels with enhanced mechanical properties.

All data support the notion that alkyltrialkoxysilane-based aerogels have huge potential for commercialization as they possess desirable properties such as flexibility, hydrophobicity, and good mechanical properties. The nature of flexible alkyltrialkoxysilanes offers additional advantages, but they also come with a higher price tag. Therefore, they are still not ready for large-scale fabrication. Although incorporating ORMOSILs in the silica backbone performed well with regard to increasing the mechanical strength, this property was only improved to a limited extent and further mechanical reinforcement is still required.

**Table 3:** Literature works reporting the synthesis of silica *aerogels and their properties*.

Precursors	Drying	Properties	Ref.
MTMS/TMOS	SCF	Monolithic, transparent (85% to visible light), and hydrophobic.	[85]
MTMS	SCF	Highly flexible and superhydrophobic (158–164°) with Young’s modulus of *0.0343–0.1411 MPa.*	[90]
MTMS	APD	Monoliths with variable pore size and pore volume, SSA 426.4 m^2^/g.	[98]
MTMS-CTAB	SCF/ APD	Transparent, hydrophobic, density of 0.15–0.24 g/cm^3^, SSA of 622 m^2^/g.	[99]
MTMS- F127	SCF/ APD	Transparent, density between 0.18 and 0.23 g/cm^3^, and SSA of 588 m^2^/g.	[99]
MTMS-CTAC	SCF	Transparent (91% light transmittance), density of 0.045 g/cm^3^, and 90% porosity.	[100]
MTMS/TEOS- CTAC	APD	SSA of 895.5 m^2^/g, hydrophobic with contact angle 153.9°.	[104]
MTMS/CTACB	APD	Low density (0.064 g/cm^3^), hydrophobic with contact angle 143.4°.	[105]
MTMS/DMDMS-CTAC	SCF	Highly flexible with density of 0.115 g/cm^3^.	[107]
MTMS/ETMS	APD/ SCF	Hydrophobic with contact angle of 142° and SSA of 416.3 m^2^/g.	[113]
MTMS/VTES	APD	Hyperelastic, hydrophobic (146°), SSA of 321 m^2^/g with compressive stress of 0.571.	[116]
MTMS/GPTMS- CTAC	SCF	Flexible, SSA of 410 m^2^/g, thermal conductivity of 0.0388 W/m K, Young’s modulus of 0.46 MPa.	[117]
MTES	SCF	Monolithic, superhydrophobic with contact angle 163°.	[121]
MTES	APD	Monolithic with SSA of 727 m^2^/g.	[123]
MTES-CTAB	SCF	Flexible, transparent, thermal conductivity of 0.0215. W/mK, hydrophobic with contact angle 151°.	[125]
ETMS-EH-208	SCF	Flexible, transparent with SSA of 383 m^2^/g.	[127]
VTMS- EH-208	SCF	Transparent, flexible, SSA of 510 m^2^/g, and thermal conductivity of 15.3 mW/mK.	[127]
PTES/TEOS-CTAB	APD	Monolithic, superhydrophobic with SSA of 215 m^2^/g and modulus of 0.55 MPa.	[128]
TEOS/PDMS	SCF	Transparent with SSA of 1200 m^2^/g.	[77]
TEOS/POSS	SCF	Density of 0.083–0.11 g/cm, 6-fold increase in compressive modulus (*0.8–3.2 MPa) with* SSA of 597–695 m^2^/g.	[133]
MPTMS/POSS	APD	Density (0.34–0.41 g/cm^3^), superhydrophobic (153°), SSA of 542–834 m^2^/g, Young’s modulus between *18.79* and 25.84 MPa.	[134]
VMDMS	APD	Superflexible, low density (0.16–0.22 g/cm), high transparency, and thermal conductivity between 15.0 and 15.4 mW/mK.	[136]
VTMS	APD	Density of 0.13–0.22 g/cm^3^, SSA of 965–1029 m^2^/g, and thermal conductivity of 15 mW/mK.	[137]
ATMS	APD	Density of 0.18–0.22 g/cm, SSA of 1021–1059 m^2^/g, and thermal conductivity of 15.3 mW/mK.	[137]
VMDMS	APD	SSA of 716–950 m^2^/g, density between 0.13 and 0.23 g/cm^3^, and thermal conductivity of 14.5–15.4 mW/mK.	[137]
AMDMS	APD	Density between 0.23 and 0.26 g/cm^3^, SSA of 766–778 m^2^/g, and thermal conductivity of 16.4 mW/mK.	[137]
VDMMS	FD	Low density, highly porous structure, superflexible in compression and bending, superhydrophobicity (157^o^).	[138]
VDMMS/VMDMS	APD	Low density (0.19–0.20 g/cm^3^), thermal conductivity (16.2–17.6 mW/mK).	[138]
VMDMS	SCF	Thermal conductivity of 14.69 mW/mK, SSA of 1218 m^2^/g with density of 0.177 g/cm^3^.	[140]
PVMDMS/MTMS	SCF	High SSA (1479 m^2^/g) with low thermal conductivity of 0.029 W/mK.	[141]
VTMS	SCF	Density between 0.05 and 0.22 g/cm^3^, SSA of 316 m^2^/g, elastic modulus ranging from 0.5 to 13.8 MPa.	[142]
GPTMS/BF_3_OET_2_	SCF	Flexible with low density (0.05–0.22 g/cm^3^), SSA of 124–570 m^2^/g, and thermal conductivity of 15.9 mW/mK.	[143]

#### 1.2.2. Bridged Polysilsesquioxanes

**Figure 10:**
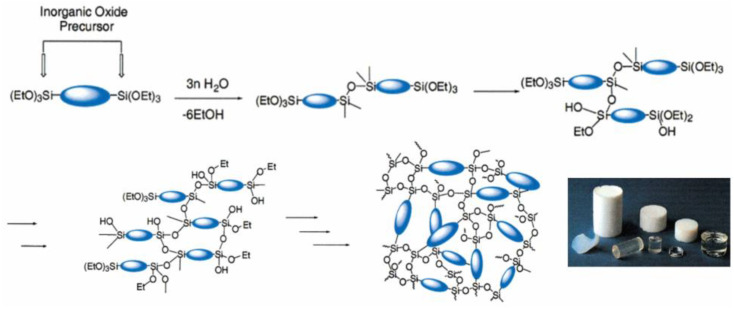
Schematic representation of synthesis of bridged silsesquioxanes. Reproduced with permission from Ref. [153]. Copyright 2001 American Chemical Society.

**Scheme 5:**
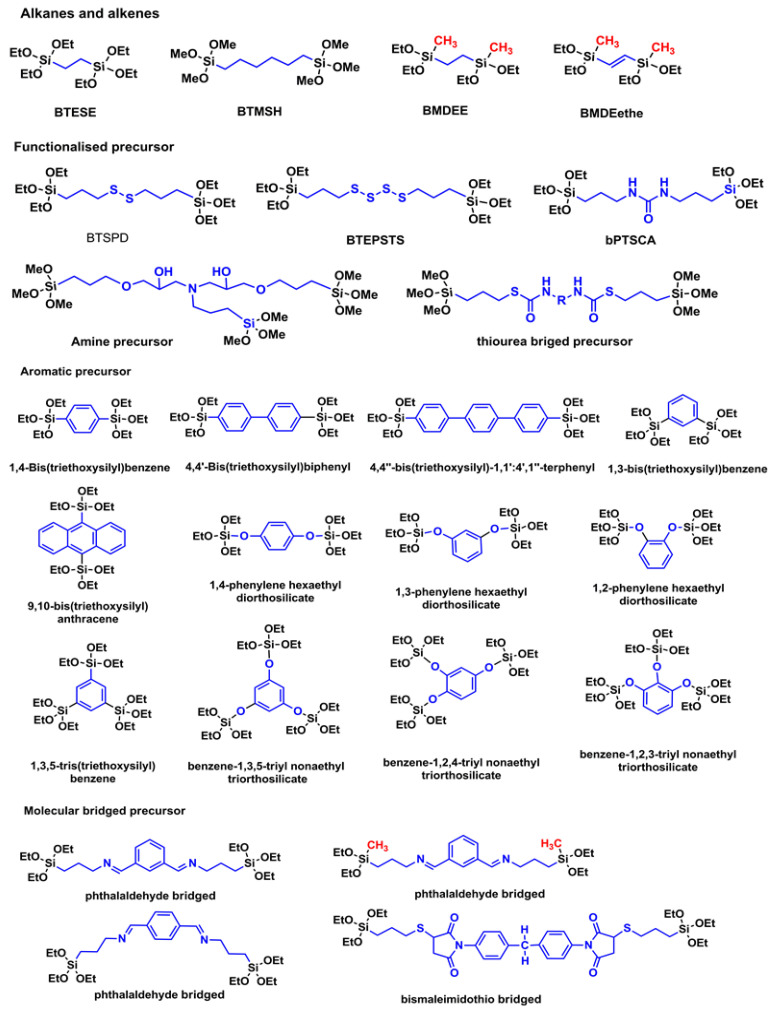
Structure of monomers used to prepare bridged polysilsesquioxanes aerogels.

Bridged polysilsesquioxane (BPS) is a class of highly cross-linked organic–inorganic hybrid materials prepared from molecular precursors ((R’O)_3_Si-R-Si(OR’)_3_, where R is typically alkyl or aryl) [144]. These precursors contain variable organic bridging groups attached to two or more trifunctional silyl groups (Scheme 5). The bridging group allows the synthesis of final materials with tunable physical, chemical, and mechanical properties [145]. BPS networks (O_1_._5_Si-R-SiO_1_._5_)_n_ with periodic mesopores are known as periodic mesoporous organosilicas (PMOs) [146,147,148]. The organic linkers will increase separation between Si atoms and further decrease cross-linking density. A typical schematic presentation of BPS is illustrated in Figure 10. BPS aerogels exhibit distinct properties such as high flexibility, light weight, and low thermal conductivity. Therefore, they are used as adsorbents for cleaning up organic contaminants [149], catalyst supports [150], low dielectrics materials [151], etc. However, in recent decades, BPS has been reported with different applications ranging from catalyst to drug delivery. Loy and Shea have extensively explored BPS using aliphatic and aromatic bridging groups in order to tailor the physical properties of the resulting aerogels [152,153,154,155]. These studies focused on porosity, relative surface area, and reactivity of the precursor.

Loy and coworkers prepared alkylene-bridged polysilsesquioxane using various bis(triethoxysilyl) alkanes and studied the effect of catalysts and the length of alkylene bridging group [156,157]. They concluded that base-catalyzed materials had higher degrees of condensation and were more hydrophobic than those prepared under acidic conditions. Among typical alkoxysilanes 1,6-bis(trimethoxysilyl)hexane (BTMSH) with a relatively long and flexible bridging group was an attractive candidate that imparted flexibility to the polysiloxane-based network [158]. Preparations of hexylene-bridged polysilsesquioxane aerogels and xerogels with a high degree of condensation and low bulk density were reported [159,160]. The resulting opaque aerogels exhibited improved flexural strength.

For many applications, it is desirable to have flexible materials. Aoki et al. introduced hexylene-bridged polysilsesquioxane to prepare flexible, transparent aerogels from BTMSH in dimethylformamide (DMF) solution followed by SCF drying [161]. The DMF solvent prevents phase separation of BTMSH-derived condensates in the course of gelation. Although the resulting aerogels showed good flexibility, an insufficient spring-back behavior upon compression was observed, which can be attributed to the remaining silanol groups on the pore surface. Surface modification with HMDZ was performed to minimize the residual silanol groups. The resulting gels dried under ambient pressure exhibited high transparency (71% transmission) with low density (0.13 g/cm^3^). Meador et al. explored porous, hydrophobic aerogels using a mixture of bis [3-(triethoxysilyl)propyl]disulfide (BTSPD), TMOS, and VTMS. Aerogels prepared using the optimum BTSPD concentration provided excellent elastic recovery at all TMOS concentrations [162]. The soft disulfide segments, which act as an organic spacer, endow the aerogels with excellent elasticity, which recovers nearly completely after a compression of 75%.

**Figure 11:**
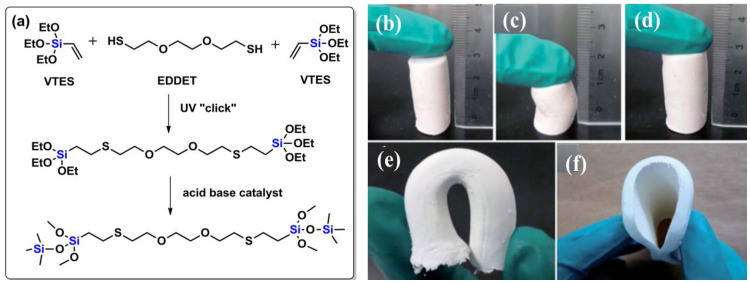
(**a**) Synthesis of bridged silsesquioxane aerogels from VTES and EDDET, (**b**–**d**) BSA compressed by finger pressure demonstrating full recovery, (**e**,**f**) BSAs retaining their integrity while being bent to 180°. Reproduced with permission from Ref. [165]. Copyright 2015 Royal Society of Chemistry.

Wang et al. produced soft and durable aerogels from bridged precursors containing C–S bonds using MPTMS and VTMS precursors *by employing* UV-initiated thiol-ene click reaction [163,164]. The alkoxy groups of the precursor influence the performance of aerogels and the flexible thioether bridge contributes to robustness. Recently, Guo and coworkers demonstrated highly flexible BSAs by introducing C–O and C–S bonds into the molecular chain using VTES and 2,2′-(ethylenedioxy)-diethanethiol (EDDET) as co-precursors followed by ambient pressure drying [165]. The BSA aerogels were easily compressed and recovered their original shape. Furthermore, the aerogels retained their integrity even after bending at 180^o^, as seen from Figure 11. In addition, these materials showed excellent repeatable absorption for organic liquids.

To investigate the relationship between catalytic method and properties of aerogels, Wang and coworkers fabricated amine-bridged polymethylsiloxane using APTMS and 3-(2,3-epoxypropoxy) propyltrimethoxysilane (EPTMS) precursor based on reactions between epoxy and amine groups [166]. The BSQ aerogels obtained exhibited low density (0.22 g/cm^3^) and high compression modulus (20.4 MPa). Recently, molecular bridged silica aerogels (MBSAs) were prepared to study the influence of catalysts using *N*,*N*-bis(propyltriethoxysilyl)carbamide (bPTSCA), which was synthesized from APTES and 3-isocyanatopropyltriethoxysilane (IPTES) using different catalysts (HCl, NH_4_OH, and NH_4_F) [167]. They observed that urethane-bridged silica aerogels showed adjustable mechanical properties ranging from rigid to elastic by varying their density. To improve the thermal stability of BSA, it was necessary to introduce thermally stable groups as bridging groups. In this regard, Zou et al. developed thiourethane-bridged polysilsesquioxanes from MPTMS via triethylamine initiated thiol-isocyanate reaction, the sulfur analog of urethane with exceptional elastic properties [168]. Three types of diisocyanates were used to investigate the influence of the rigidity of the bridging groups. The resulting aerogels exhibited low density, low thermal conductivity, and good mechanical properties to withstand 50% deformation under compression.

**Figure 12:**
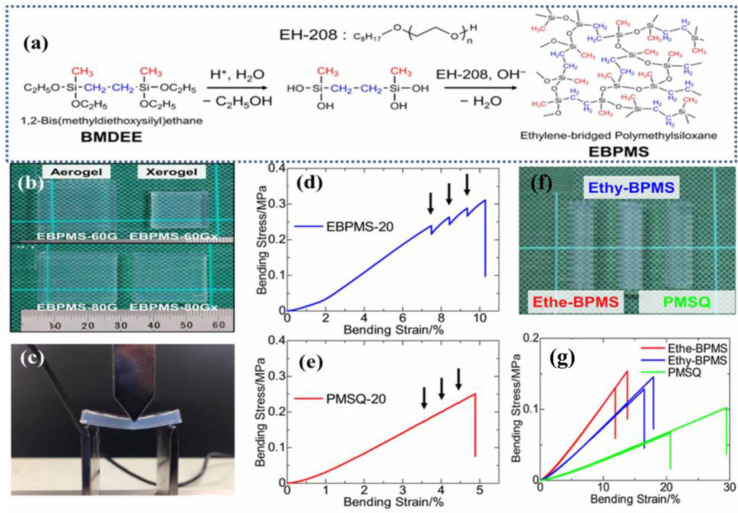
(**a**) Synthesis of EBPMS aerogel, (**b**) transparent EBPMS aerogel and xerogels, (**c**) bending deformation of EBPMS aerogel, (**d**,**e**) stress–strain curves obtained from three-point bending tests on EBPMS and PMSQ aerogels. (**f**,**g**) Appearance and three-point bending tests of Ethy-BPMS aerogels. Reproduced with permission from Refs. [169,170]. Copyright 2016, 2017 American Chemical Society.

An ethylene-bridged polymethylsiloxane (EBPMS) network has been investigated. EBPMS is analogous to the PMSQ network, except that one-third of the siloxane oxygens are substituted with a bridging ethylene group [169]. Schematic presentation of the synthesis of EBPMS aerogels from 1,2-bis(methoxydithoxysilyl)ethane (BMDEE) using EH-208 surfactant is shown in Figure 12. Compared with PMSQ, the EBPMS aerogels exhibit higher bending stress and strain. This enhanced tolerance of the EBPMS against bending reflects the effect of substituted ethylene groups. As observed from Figure 12d, the slope of the stress–strain curves dramatically decrease with an increase in bending strain. In order to study the influence of organic bridging groups on the mechanical properties, Shimizu et al. further fabricated transparent ethylene (CH_2_-CH_2_) and ethenylene (CH=CH)-bridged polymethylsiloxane (Ethy-BPMS) aerogels focusing on bulk density and mechanical properties [170]. The Ethy-BPMS aerogel was composed of a network similar to that of PMSQ, except for a partial substitution of the ethylene bridge for the oxygen bridge in the siloxane bond. The Ethy-BPMS aerogels obtained were more flexible than PMSQ aerogels. The curves obtained from three-point bending tests show that Ethe-BPMS and Ethy-BPMS both exhibited different flexural moduli but similar bending strength. In addition, the compressive modulus of the bridged aerogels was much higher than that of PMSQ aerogels (Figure 12h). The results suggest that the molecular structure of the cross-linked network affects the macroscopic mechanical properties of the aerogels. Table 4 presents a summary of the literature related to the properties of BSQ aerogels.

**Figure 13:**
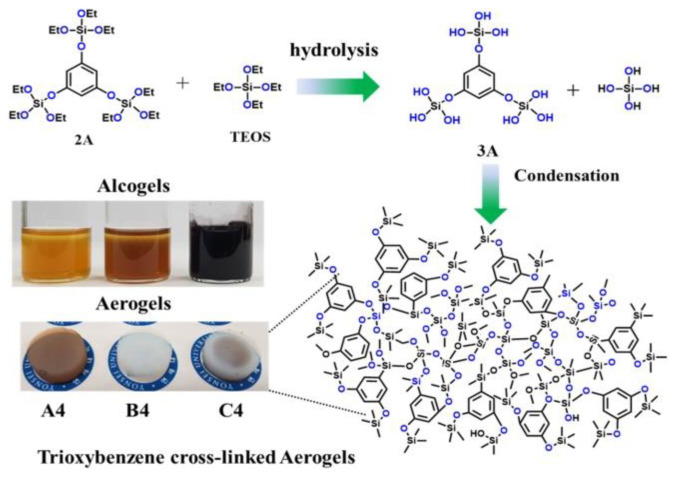
Synthesis of trioxybenzene cross-linked composite aerogels. Reprinted with permission from Ref. [173]. Copyright 2020 Elsevier.

In another report, Schaefer et al. evaluated arylene-bridged polymethylsiloxane by inserting organic rigid-rod spacers at regular intervals into the silicate network, which formed an integral part of the chemical connectivity of the material [171]. They obtained highly porous aerogels with high surface areas (1880 m^2^/g) benefiting from the rigid arylene bridge. Aromatic containing bridges are stiffer, but provide excellent control over pore size and the distribution of pores. Because of the stiff bridge, the phenylene-bridged BSQ synthesized by Boday et al. had a flexural strength of 0.048 MPa, which is stronger than that of hexylene-bridged aerogels and 30% stronger than that of silica aerogels of the same density [159]. More recently, our group synthesized multi-functional dihydroxy- and trihydroxybenzene-bridged silica aerogels using various isomers [172,173]. A schematic presentation of the synthesis of trioxybenzene cross-linked silica aerogels is shown in Figure 13. The obtained aerogels exhibited improved mechanical strength, high surface area (1150–1250 m^2^/g), high porosity (96%), low density (0.024–0.06 g/cm^3^), and low thermal conductivity in the range of 0.033–0.06 W/mK. In addition, these bridged aerogels showed hydrophobic natures without the use of silylating reagents.

**Figure 14:**
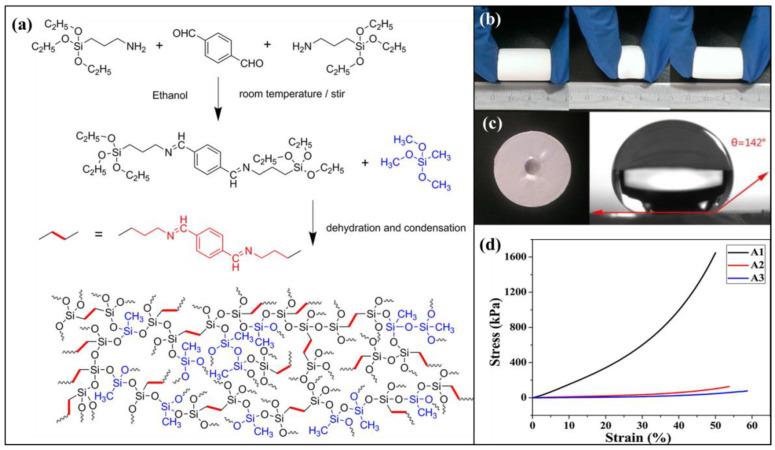
(**a**) Synthesis of MBS aerogels from TPAL, APTES, and MTMS co-precursors, (**b**) BSQ aerogel compressed by fingers, (**c**) water droplet on the aerogel surface, (**d**) stress–strain curves of MBS aerogels. Reproduced with permission from Ref. [175]. Copyright 2018 American Chemical Society.

Molecular-bridged silica aerogels (MBSA) are good candidates for oil–water separation. MBSAs were synthesized based on the catalyst-free bridging of APTES and terephthalaldehyde (TPAL) via Schiff base condensation through a one-pot autocatalytic approach [174]. The aerogels obtained exhibited good mechanical properties with 90% deformation and high absorption capability (11–24 times their own weight). Recently, Chen et al. developed a facile method to prepare MBSAs using TPAL, APTES, and MTMS co-precursors in the presence of acetic acid as catalyst followed by vacuum during. The resulting monolithic aerogels exhibited low density and excellent flexibility, with a Young’s modulus of 0.029 MPa (Figure 14) [175]. They also showed good absorption for different organic liquids. To further study the influence of precursor and solvent, Chen and coworkers synthesized two types of aerogels by reacting m-phthalaldehyde (MPA) with APTES and APDEMS dried at ambient pressure [176]. MPA/APTE-based aerogels were hydrophilic with small particle sizes. In contrast, MPA/APDEMS-based aerogels were hydrophobic with large particle sizes and thick networks due to the presence of methyl groups and slow reaction rates. Recently, Tang et al. prepared bismaleimide-bridged silsesquioxane (BMIT-BSA) by utilizing MPTES and (4,4′-diphenylmethylene)bismaleimide (BMI) via thiol-maleimide click reaction followed by vacuum drying [177]. The aerogels obtained showed excellent heat resistance (0.044 W/mK), were superhydrophobic, light-weight, and had enhanced thermal stability (336 °C).

Incorporating flexible, organic linking groups into the silica backbone has been shown to be a versatile way to improve the elastic properties of aerogels. The aerogels can recover from compression up to as much as 50% strain and are flexible in some cases. The flexible linking groups also result in greater hydrophobicity and provide a means to tailor pore structure. Use of flexible polymer-cross-linked aerogels is a promising route to making robust aerogel monoliths, thin films, and sheets, enabling a multitude of aerospace applications.

**Table 4:** Literature studies on bridged silsesquioxane aerogels.

Precursor	Drying	Properties	Ref.
BTSHM	SCF	Density of 0.093 g/cm^3^, SSA of 778 m^2^/g with Young’s modulus 0.079 MPa.	[159]
BTSHM	SCF	Transparent, low density (0.14–0.22 g/cm^3^), SSA of 874 m^2^/g.	[161]
BTSHM-HMDS	APD	Flexible, transparent, low density (0.13 g/cm^3^), 90% porosity with SSA of 924 m^2^/g.	[161]
BTSPD/TMOS/VTMS	SCF	Density of 0.126–0.257 g/cm^3^, porosity (86–94.8%), SSA between 3.42 and 718 m^2^/g, and Young’s modulus 0.5–3.7 MPa.	[162]
MPTMS/VTMS	VD	Density ranging from 0.064 to 0.085 g/cm^3^, SSA of 338–363 m^2^/g, and Young’s modulus of 29.4–117.2 kPa.	[163]
EDDET/VTES	APD	Density of 0.125–0.237 g/cm^3^_,_ SSA between 58 and 135 m^2^/g, thermal conductivity of 0.037–0.043, Young’s modulus 0.11–1.31 MPa.	[165]
Amine-bridged	SCF	Density of 0.1–0.22 g/cm^3^, SSA of 321–416 m^2^/g, Young’s modulus 3.1–20.4 MPa.	[166]
Urethane-bridged	SCF	Density of 0.049–0.59 g/cm^3^, SSA of 157–472 m^2^/g, with Young’s modulus of 0.028–34 MPa.	[167]
Thiourethane-bridged	VD	Density of 0.05–0.082 g/cm^3^, thermal conductivity of 0.018–0.021 W/mK, and Young’s modulus of 0.004–0.43 MPa.	[168]
Ethylene-bridged	SCF	Transparent, density of 0.19 g/cm^3^ with SSA of 599–797 m^2^/g.	[169]
Ethenylene-bridged	SCF	Transparent, low density (0.14 g/cm^3^) with SSA of 946 m^2^/g.	[170]
1,4-phenylene-bridged	SCF	Density of 0.097 g/cm^3^, SSA of 808 m^2^/g with Young’s modulus 0.553 Mpa.	[159]
1,4-phenylene-bridged	SCF	SSA of 958–1670 m^2^/g with density of 0.08–0.064 g/cm^3^.	[171]
4,4′-biphenylene-bridged	SCF	Density of 0.71 g/cm^3^.	[171]
1,3,5-phenylene-bridged	SCF	Density of 0.28 g/cm^3^, SSA of 756 m^2^/g.	[171]
TPAL/APTES	SCF	Low density (0.053–0.07 g/cm^3^) with SSA of 38.7–57.9 m^2^/g.	[174]
TPAL/APTES/MTMS	VD	Flexible, SSA of 16.8–91.2 m^2^/g, density of 0.71–0.21 g/cm^3^ with Young’s modulus of 0.029 MPa.	[175]
MPA/APTES	APD	Low density (0.112–0.149 g/cm^3^) with Young’s modulus of 0.12–0.16 MPa.	[176]
MPA/APDEMS	APD	Density of 0.08–0.064 g/cm^3^ with Young’s modulus of 1.12–1.14 MPa, thermal conductivity of 0.051–0.058 W/mK.	[176]
MPTES/BMI	VD	Hydrophobic, low density (0.09 g/cm^3^), thermal conductivity of 0.044 W/mK, elastic modulus of 10.3–25.8 kPa.	[177]

## 2. Current Status of Aerogels

For a long time, aerogels have been seen as curious materials existing mainly in labs. However, today silica aerogels at least, as well as some organic (polyurethane) and carbon aerogels, are commercially available and are produced in a batch-wise manner. Today, the material cost for aerogel insulation is twenty times higher than that of standard insulation products. The chances of aerogel products to be transferred to the market mainly depends on the further reduction in cost associated with the production process. Therefore, a fundamental understanding of thermodynamics and kinetics of the precursor, solvent exchange, and drying method is required. In addition, reactor design and effective use of reactor volume should be considered.

Concerning silica aerogels, sodium silicates and alkoxysilane are well-known precursors. With the increasing adoption of silica aerogels by various industries, the demand for silica aerogels is estimated to rapidly increase over the forecasted period. Silica is also found in different types of biomass (ash). Recently, ashes of various (industrial) biowastes containing silica species, such as rice husk, bagasse, oil shale, and wheat husk, have been used as a silica source for aerogel production. Green Earth Aerogels (GEAT) has already commercialized the production of silica aerogels from rice husks.

Currently, the main market application of aerogels is thermal insulation. However, further applications such as for carriers and filler materials (cosmetics, pharmaceutics) and absorbers (environmental cleanups) are already developed. Aerogels are now commercialized by several companies. The primary products are powder, granules, monoliths, and blankets (Table 5). Cabot Corporation uses aerogels as insulators for windows, the Aspen Aerogels Company as flexible insulation products, and the American Aerogel Corp. as open-cell foams. Kobel et al. provided some insight into the size of the global market for aerogels [178], which was about USD 80 million in 2008 and USD 230.82 million in 2016. Aspen Aerogels is the global leader in this industry, with a gross production of USD 115.34 million in 2016. With increasing commercialization, the silica aerogel market had a value of USD 250 million in 2020 and is likely to witness significant growth over the next seven years owing to its rising demand from various industries.

## 3. Conclusions

Aerogel synthesis is one of the major research areas in the field of materials science. A systematic literature survey revealed that there are several challenges in the commercialization of aerogels. This comprehensive review discusses the synthetic progress that has been made in the developments of aerogels and provides guidelines for finding suitable precursors for specific applications. Regardless of the chemical makeup, aerogels offer many fascinating properties. These exceptional properties have drawn a great deal of interest from researchers around the world for their use in many different applications. The use of superinsulating materials is considered a most promising market for building retrofits, where it allows a significant reduction in energy requirements for existing buildings. However, the inherent fragility, hydrophilic nature, low elasticity, and high costs associated with the supercritical drying method affect the overall production and handling of aerogels, which limits their practical applications. Despite progress toward environmental stability and drying under ambient pressure, the slow commercialization of aerogels is ultimately traced to their fragility.

To date, efforts aimed improving the mechanical properties of aerogels have been dedicated via different approaches, such as by incorporating organic monomers, polymers, and biopolymers. Additionally, by choosing cheaper precursors, the production cost of aerogels can be effectively reduced. Hybrid materials offer an unlimited potential for further development. Unfortunately, increasing organic content does not only inevitably boost inhomogeneity, but comes at the cost of a noticeable decrease in surface area and porosity. Although organic, inorganic, and composite aerogels made with different precursors each have their own advantages, none of them can fulfill all of the required properties, particularly flexibility, density, surface area, low shrinkage, moisture resistance, and thermal stability. Therefore, aerogel commercialization is still in its infancy and needs further advancement.

Overall, a viable aerogel requires synthetic parameters and precursors that reach a balance between density, porosity, flexibility, and thermal conductivity. Aerogel flexibility is highly sought, yet difficult to achieve. Regardless of their drawbacks, industrial applications of aerogels under current attention are back in line with their fundamental properties focusing on thermal insulation. The development of improved alternative methods must continue to achieve sufficient mechanical strength with low production cost and scalable drying procedures. It is to be hoped more researchers will focus on the preparation of novel single-component aerogels, material designs of composite aerogels, and industrial applications, to give this state of matter a bright future.

## Figures and Tables

**Table 5:** Primary manufacturers of aerogels. Reproduced with permission from Ref. [55]. Copyright 2017 Elsevier.

Region	Country	Manufacturer	Chemical Nature of Aerogels	Aerogel Form
Asia	Japan	Tiem Factory Inc., Tokyo	Silica (APD)	Powder, granules, and monoliths
	South Korea	JIOS Aerogel Corp, Seoul	Silica	Powder, granules, and blankets
	China	Nano High-Tech Co., Ltd., Daejeon	Silica, organic	Diverse
		Alison Hi-Tech Co., Ltd., Guangdong	Mainly silica	Blankets, granules, and panel
		Surnano Aerogel Co., Ltd., Shaoxing City	Mainly silica	Granules, sheet, and panel
	Malaysia	Gelanggang Kencana Sdn. Bhd., Kuala Lumpur	Silica	Powder
North America	USA	Aspen Aerogels, Northborough, MA	Silica incorporated in textiles	Blankets
		Aerogel Technologies, Boston, MA	Organic aerogels	Panels and particles
		Nano Pore Inc., Albuquerque, NM	Silica	Thin films and related products
		TAASI Corporation Marketech International, Phoenix, AZ	Silica	Powder and monoliths
		CDT Systems Inc., Addison, TX	Carbon, composite materials	Monoliths
		Dow Corning, Midland, MI	Silica	Powder
Europe	Germany	Cabot aerogels, Frankfurt	Silica incorporated in support matrix	Powder, blankets, sheets, and granules
	Spain	Green Earth Aerogels, Barcelona	Silica from rice husk	Powder and granules
		Enersens SAS, Rochetoirin	Silica	Powder and monoliths
		Keey Aerogels, Habsheim	Silica and silica incorporated in matrix	Particles
	Sweden	Airglass AB, Staffanstorp	Transparent silica	Monoliths and panels
		Svenska Aerogel, Gävle	Silica	Powder and adsorbents
	Switzerland	Nexaero, Berlin	Hydrophobic silica	Particles
	France	Enersens, Rochetoirin	Silica	Powder, granules, blankets, and panels
	Portugal	Active Aerogels, Coimbra	Silica composite	Powder, granules, blankets, and adsorbents
	UK	Thermablok Aerogels, Ashford	Silica	Blankets

## Data Availability

Data Availability Statements are available in section “MDPI Research Data Policies” at https://www.mdpi.com/ethics (accessed on 3 April 2023).

## References

[1] Allahbakhsh A., Bahramian A.R. (2015). Self-assembled and pyrolyzed carbon aerogels: An overview of their preparation mechanisms, properties and applications. Nanoscale.

[2] Lee J.-H., Park S.-J. (2020). Recent advances in preparations and applications of carbon aerogels: A review. Carbon.

[3] Young C., Park T., Yi J.W., Kim J., Hossain M.S.A., Kaneti Y.V., Yamauchi Y. (2018). Advanced Functional Carbons and Their Hybrid Nanoarchitectures towards Supercapacitor Applications. ChemSusChem.

[4] Wang H., Shao Y., Mei S., Lu Y., Zhang M., Sun J.K., Matyjaszewski K., Antonietti M., Yuan J. (2020). Polymer-Derived Heteroatom-Doped Porous Carbon Materials. Chem. Rev..

[5] Gan G., Li X., Fan S., Wang L., Qin M., Yin Z., Chen G. (2019). Carbon Aerogels for Environmental Clean-Up. Eur. J. Inorg. Chem..

[6] Almeida C.M.R., Ghica M.E., Durães L. (2020). An overview on alumina-silica-based aerogels. Adv. Colloid Interface Sci..

[7] Ziegler C., Wolf A., Liu W., Herrmann A.-K., Gaponik N., Eychmüller A. (2017). Modern Inorganic Aerogels. Angew. Chem. Int. Ed..

[8] Sui R., Charpentier P. (2012). Synthesis of metal oxide nanostructures by direct sol-gel chemistry in supercritical fluids. Chem. Rev..

[9] Maleki H., Durães L., Portugal A. (2014). An overview on silica aerogels synthesis and different mechanical reinforcing strategies. J. Non-Cryst. Solids.

[10] Gurav J.L., Jung I.-K., Park H.-H., Kang E.S., Nadargi D.Y. (2010). Silica Aerogel: Synthesis and Applications. J. Nanomater..

[11] Kistler S.S., Fischer E.A., Freeman I.R. (1943). Sorption and Surface Area in Silica Aerogel. J. Am. Chem. Soc..

[12] Kistler S.S. (1932). Coherent Expanded-Aerogels. J. Phys. Chem..

[13] Shewale P.M., Rao A.V., Rao A.P. (2008). Effect of different trimethyl silylating agents on the hydrophobic and physical properties of silica aerogels. Appl. Surf. Sci..

[14] Mahadik D.B., Venkateswara Rao A., Parale V.G., Kavale M.S., Wagh P.B., Ingale S.V., Gupta S.C. (2011). Effect of surface composition and roughness on the apparent surface free energy of silica aerogel materials. Appl. Phys. Lett..

[15] Wagh P.B., Ingale S.V., Gupta S.C. (2010). Comparison of hydrophobicity studies of silica aerogels using contact angle measurements with water drop method and adsorbed water content measurements made by Karl Fischer’s titration method. J. Sol-Gel Sci. Technol..

[16] Rao A.V. (2018). Elastic superhydrophobic and water glass-based silica aerogels and applications. J. Sol-Gel Sci. Technol..

[17] Bangi U.K., Venkateswara Rao A., Parvathy Rao A. (2008). A new route for preparation of sodium-silicate-based hydrophobic silica aerogels via ambient-pressure drying. Sci. Technol. Adv. Mater..

[18] Bhagat S.D., Kim Y.-H., Ahn Y.-S., Yeo J.-G. (2007). Rapid synthesis of water-glass based aerogels by in situ surface modification of the hydrogels. Appl. Surf. Sci..

[19] Lee C.J., Kim G.S., Hyun S.H. (2002). Synthesis of silica aerogels from waterglass via new modified ambient drying. J. Mater. Sci..

[20] Nah H.Y., Kim Y., Kim T., Lee K.Y., Parale V.G., Lim C.H., Seo J.Y., Park H.H. (2020). Comparisonal studies of surface modification reaction using various silylating agents for silica aerogel. J. Sol-Gel Sci. Technol..

[21] Gurav J.L., Rao A.V., Rao A.P., Nadargi D.Y., Bhagat S.D. (2009). Physical properties of sodium silicate based silica aerogels prepared by single step sol–gel process dried at ambient pressure. J. Alloys Compd..

[22] Parvathy Rao A., Venkateswara Rao A. (2010). Modifying the surface energy and hydrophobicity of the low-density silica aerogels through the use of combinations of surface-modification agents. J. Mater. Sci..

[23] Bangi U.K.H., Parvathy Rao A., Hirashima H., Venkateswara Rao A. (2009). Physico-chemical properties of ambiently dried sodium silicate based aerogels catalyzed with various acids. J. Sol-Gel Sci. Technol..

[24] Rao A.P., Rao A.V. (2009). Improvement in optical transmission of the ambient pressure dried hydrophobic nanostructured silica aerogels with mixed silylating agents. J. Non-Cryst. Solids.

[25] Bhagat S.D., Kim Y.-H., Ahn Y.-S., Yeo J.-G. (2006). Textural properties of ambient pressure dried water-glass based silica aerogel beads: One day synthesis. Microporous Mesoporous Mater..

[26] Bhagat S.D., Kim Y.-H., Moon M.-J., Ahn Y.-S., Yeo J.-G. (2007). A cost-effective and fast synthesis of nanoporous SiO_2_ aerogel powders using water-glass via ambient pressure drying route. Solid State Sci..

[27] Bhagat S.D., Kim Y.-H., Suh K.-H., Ahn Y.-S., Yeo J.-G., Han J.-H. (2008). Superhydrophobic silica aerogel powders with simultaneous surface modification, solvent exchange and sodium ion removal from hydrogels. Microporous Mesoporous Mater..

[28] Sarawade P.B., Kim J.-K., Hilonga A., Kim H.T. (2010). Production of low-density sodium silicate-based hydrophobic silica aerogel beads by a novel fast gelation process and ambient pressure drying process. Solid State Sci..

[29] Bangi U.K.H., Pandit S.S., Bagal D.B., Park H.H. (2019). Preparation of Sodium Silicate–Based Aerogels Using a Two-Step Sol–Gel Process and Ambient Pressure Drying. Macromol. Symp..

[30] La D.D., Bhosale S.V., Jones L.A., Bhosale S.V. (2018). Tetraphenylethylene-Based AIE-Active Probes for Sensing Applications. ACS Appl. Mater. Interfaces.

[31] Tillotson T.M., Hrubesh L.W. (1992). Transparent ultralow-density silica aerogels prepared by a two-step sol-gel process. J. Non-Cryst. Solids.

[32] Loy D.A., Russick E.M., Yamanaka S.A., Baugher B.M., Shea K.J. (1997). Direct Formation of Aerogels by Sol−Gel Polymerizations of Alkoxysilanes in Supercritical Carbon Dioxide. Chem. Mater..

[33] Folgar C., Folz D., Suchicital C., Clark D. (2007). Microstructural evolution in silica aerogel. J. Non-Cryst. Solids.

[34] Mahadik D.B., Lee Y.K., Chavan N.K., Mahadik S.A., Park H.-H. (2016). Monolithic and shrinkage-free hydrophobic silica aerogels via new rapid supercritical extraction process. J. Supercrit. Fluids.

[35] Tamon H., Kitamura T., Okazaki M. (1998). Preparation of Silica Aerogel from TEOS. J. Colloid Interface Sci..

[36] Cai H.F., Jiang Y.G., Feng J., Zhang S.Z., Peng F., Xiao Y.Y., Li L.J., Feng J.Z. (2020). Preparation of silica aerogels with high temperature resistance and low thermal conductivity by monodispersed silica sol. Mater. Des..

[37] Hegde N.D., Venkateswara Rao A. (2006). Organic modification of TEOS based silica aerogels using hexadecyltrimethoxysilane as a hydrophobic reagent. Appl. Surf. Sci..

[38] Bangi U.K.H., Park C.-S., Baek S., Park H.-H. (2012). Improvement in optical and physical properties of TEOS based aerogels using acetonitrile via ambient pressure drying. Ceram. Int..

[39] Venkateswara Rao A., Parvathy N.N. (1993). Effect of gel parameters on monolithicity and density of silica aerogels. J. Mater. Sci..

[40] Venkateswara Rao A., Bhagat S.D. (2004). Synthesis and physical properties of TEOS-based silica aerogels prepared by two step (acid–base) sol–gel process. Solid State Sci..

[41] Pisal A.A., Rao A.V. (2016). Comparative studies on the physical properties of TEOS, TMOS and Na_2_SiO_3_ based silica aerogels by ambient pressure drying method. J. Porous Mater..

[42] Estok S.K., Hughes T.A., Carroll M.K., Anderson A.M. (2014). Fabrication and characterization of TEOS-based silica aerogels prepared using rapid supercritical extraction. J. Sol-Gel Sci. Technol..

[43] Hæreid S., Dahle M., Lima S., Einarsrud M.A. (1995). Preparation and properties of monolithic silica xerogels from TEOS-based alcogels aged in silane solutions. J. Non-Cryst. Solids.

[44] Rao A.P., Rao A.V., Pajonk G.M. (2005). Hydrophobic and Physical Properties of the Two Step Processed Ambient Pressure Dried Silica Aerogels with Various Exchanging Solvents. J. Sol-Gel Sci. Technol..

[45] Wei T.-Y., Chang T.-F., Lu S.-Y., Chang Y.-C. (2007). Preparation of Monolithic Silica Aerogel of Low Thermal Conductivity by Ambient Pressure Drying. J. Am. Ceram. Soc..

[46] Smith D.M., Deshpande R., Jeffrey Brinke C. (1992). Preparation of Low-Density Aerogels at Ambient Pressure. MRS Proc..

[47] Prakash S.S., Brinker C.J., Hurd A.J., Rao S.M. (1995). Silica aerogel films prepared at ambient pressure by using surface derivatization to induce reversible drying shrinkage. Nature.

[48] Hilonga A., Kim J.-K., Sarawade P.B., Kim H.T. (2009). Low-density TEOS-based silica aerogels prepared at ambient pressure using isopropanol as the preparative solvent. J. Alloys Compd..

[49] Mahadik D.B., Rao A.V., Kumar R., Ingale S.V., Wagh P.B., Gupta S.C. (2011). Reduction of processing time by mechanical shaking of the ambient pressure dried TEOS based silica aerogel granules. J. Porous Mater..

[50] Mahadik D.B., Rao A.V., Rao A.P., Wagh P.B., Ingale S.V., Gupta S.C. (2011). Effect of concentration of trimethylchlorosilane (TMCS) and hexamethyldisilazane (HMDZ) silylating agents on surface free energy of silica aerogels. J. Colloid. Interface Sci..

[51] Çok S.S., Gizli N. (2020). Hydrophobic silica aerogels synthesized in ambient conditions by preserving the pore structure via two-step silylation. Ceram. Int..

[52] Xie Z., Henderson E.J., Dag O., Wang W., Lofgreen J.E., Kubel C., Scherer T., Brodersen P.M., Gu Z.Z., Ozin G.A. (2011). Periodic mesoporous hydridosilica--Synthesis of an “impossible” material and its thermal transformation into brightly photoluminescent periodic mesoporous nanocrystal silicon-silica composite. J. Am. Chem. Soc..

[53] Moitra N., Kanamori K., Shimada T., Takeda K., Ikuhara Y.H., Gao X., Nakanishi K. (2013). Synthesis of Hierarchically Porous Hydrogen Silsesquioxane Monoliths and Embedding of Metal Nanoparticles by On-Site Reduction. Adv. Funct. Mater..

[54] Woignier T., Primera J., Alaoui A., Etienne P., Despestis F., Calas-Etienne S. (2015). Mechanical Properties and Brittle Behavior of Silica Aerogels. Gels.

[55] Amonette J.E., Matyáš J. (2017). Functionalized silica aerogels for gas-phase purification, sensing, and catalysis: A review. Microporous Mesoporous Mater..

[56] Kistler S.S. (1931). Coherent Expanded Aerogels and Jellies. Nature.

[57] Schottner G. (2001). Hybrid Sol−Gel-Derived Polymers:  Applications of Multifunctional Materials. Chem. Mater..

[58] Sanchez C., Belleville P., Popall M., Nicole L. (2011). Applications of advanced hybrid organic–inorganic nanomaterials: From laboratory to market. Chem. Soc. Rev..

[59] Sanchez C., Julián B., Belleville P., Popall M. (2005). Applications of hybrid organic–inorganic nanocomposites. J. Mater. Chem..

[60] Kanamori K., Klein L., Aparicio M., Jitianu A. (2018). Hybrid Aerogels. Handbook of Sol-Gel Science and Technology: Processing, Characterization and Applications.

[61] Boury B., Corriu R.J.P. (2002). Auto-organisation of hybrid organic–inorganic materials prepared by sol–gel chemistry. Chem. Commun..

[62] Li H., Song L., Fu Y., Wei Y., Li R., Liu H. (2017). Loads transfer across static electrical phase interfaces in silica aerogel/polymethyl methacrylate composites. Compos. Sci. Technol..

[63] Leventis N., Sotiriou-Leventis C., Zhang G., Rawashdeh A.-M.M. (2002). Nanoengineering Strong Silica Aerogels. Nano Lett..

[64] Shimojima A., Kuroda K. (2006). Designed synthesis of nanostructured siloxane–organic hybrids from amphiphilic silicon-based precursors. Chem. Rec..

[65] Kanamori K., Nakanishi K. (2011). Controlled pore formation in organotrialkoxysilane-derived hybrids: From aerogels to hierarchically porous monoliths. Chem. Soc. Rev..

[66] Kanamori K. (2011). Organic–inorganic hybrid aerogels with high mechanical properties via organotrialkoxysilane-derived sol–gel process. J. Ceram. Soc. Jpn..

[67] Baney R.H., Itoh M., Sakakibara A., Suzuki T. (1995). Silsesquioxanes. Chem. Rev..

[68] Kanamori K. (2014). Monolithic silsesquioxane materials with well-defined pore structure. J. Mater. Res..

[69] Cordes D.B., Lickiss P.D., Rataboul F. (2010). Recent Developments in the Chemistry of Cubic Polyhedral Oligosilsesquioxanes. Chem. Rev..

[70] Tanaka K., Ishiguro F., Chujo Y. (2010). POSS Ionic Liquid. J. Am. Chem. Soc..

[71] Kamino B.A., Bender T.P. (2013). The use of siloxanes, silsesquioxanes, and silicones in organic semiconducting materials. Chem. Soc. Rev..

[72] Chinnam P.R., Wunder S.L. (2011). Polyoctahedral Silsesquioxane-Nanoparticle Electrolytes for Lithium Batteries: POSS-Lithium Salts and POSS-PEGs. Chem. Mater..

[73] Chua Y.T., Lin C.X.C., Kleitz F., Zhao X.S., Smart S. (2013). Nanoporous organosilica membrane for water desalination. Chem. Commun..

[74] Castricum H.L., Paradis G.G., Mittelmeijer-Hazeleger M.C., Kreiter R., Vente J.F., ten Elshof J.E. (2011). Tailoring the Separation Behavior of Hybrid Organosilica Membranes by Adjusting the Structure of the Organic Bridging Group. Adv. Funct. Mater..

[75] Lebeau B., Innocenzi P. (2011). Hybrid materials for optics and photonics. Chem. Soc. Rev..

[76] Novak B.M., Auerbach D., Verrier C. (1994). Low-Density, Mutually Interpenetrating Organic-Inorganic Composite Materials via Supercritical Drying Techniques. Chem. Mater..

[77] Kramer S.J., Rubio-Alonso F., Mackenzie J.D. (1996). Organically Modified Silicate Aerogels, “Aeromosils”. MRS Proc..

[78] Nakanishi K., Kanamori K. (2005). Organic–inorganic hybrid poly(silsesquioxane) monoliths with controlled macro- and mesopores. J. Mater. Chem..

[79] Loy D.A., Baugher B.M., Baugher C.R., Schneider D.A., Rahimian K. (2000). Substituent Effects on the Sol−Gel Chemistry of Organotrialkoxysilanes. Chem. Mater..

[80] Schwertfeger F., Glaubitt W., Schubert U. (1992). Hydrophobic aerogels from Si(OMe)_4_/MeSi(OMe)_3_ mixtures. J. Non-Cryst. Solids.

[81] Hüsing N., Schwertfeger F., Tappert W., Schubert U. (1995). Influence of supercritical drying fluid on structure and properties of organically modified silica aerogels. J. Non-Cryst. Solids.

[82] Hüsing N., Schubert U., Misof K., Fratzl P. (1998). Formation and Structure of Porous Gel Networks from Si(OMe)_4_ in the Presence of A(CH_2_)nSi(OR)_3_ (A = Functional Group). Chem. Mater..

[83] Hüsing N., Schubert U., Mezei R., Fratzl P., Riegel B., Kiefer W., Kohler D., Mader W. (1999). Formation and Structure of Gel Networks from Si(OEt)_4_/(MeO)_3_Si(CH_2_)_3_NR’_2_ Mixtures (NR’_2_ = NH_2_ or NHCH_2_CH_2_NH_2_). Chem. Mater..

[84] Dong H., Zhang Z., Lee M.-H., Mueller D.W., Reidy R.F. (2007). Sol-gel polycondensation of methyltrimethoxysilane in ethanol studied by 29Si NMR spectroscopy using a two-step acid/base procedure. J. Sol-Gel Sci. Technol..

[85] Venkateswara Rao A., Haranath D. (1999). Effect of methyltrimethoxysilane as a synthesis component on the hydrophobicity and some physical properties of silica aerogels. Microporous Mesoporous Mater..

[86] Venkateswara Rao A., Pajonk G.M. (2001). Effect of methyltrimethoxysilane as a co-precursor on the optical properties of silica aerogels. J. Non-Cryst. Solids.

[87] Rao A.V., Pajonk G.M., Bhagat S.D., Barboux P. (2004). Comparative studies on the surface chemical modification of silica aerogels based on various organosilane compounds of the type RnSiX4−n. J. Non-Cryst. Solids.

[88] Hegde N.D., Venkateswara Rao A. (2007). Physical properties of methyltrimethoxysilane based elastic silica aerogels prepared by the two-stage sol–gel process. J. Mater. Sci..

[89] Bhagat S.D., Oh C.-S., Kim Y.-H., Ahn Y.-S., Yeo J.-G. (2007). Methyltrimethoxysilane based monolithic silica aerogels via ambient pressure drying. Microporous Mesoporous Mater..

[90] Rao A.V., Bhagat S.D., Hirashima H., Pajonk G.M. (2006). Synthesis of flexible silica aerogels using methyltrimethoxysilane (MTMS) precursor. J. Colloid. Interface Sci..

[91] Borzęcka N.H., Nowak B., Gac J.M., Głaz T., Bojarska M. (2020). Kinetics of MTMS-based aerogel formation by the sol-gel method-experimental results and theoretical description. J. Non-Cryst. Solids.

[92] Yun S., Guo T., Zhang J., He L., Li Y., Li H., Zhu X., Gao Y. (2017). Facile synthesis of large-sized monolithic methyltrimethoxysilane-based silica aerogel via ambient pressure drying. J. Sol-Gel Sci. Technol..

[93] Rao A.V., Kalesh R.R., Pajonk G.M. (2003). Hydrophobicity and physical properties of TEOS based silica aerogels using phenyltriethoxysilane as a synthesis component. J. Mater. Sci..

[94] Venkateswara Rao A., Hegde N.D., Shewale P.M. (2007). Imperviousness of the hydrophobic silica aerogels against various solvents and acids. Appl. Surf. Sci..

[95] Martín L., Ossó J.O., Ricart S., Roig A., García O., Sastre R. (2008). Organo-modified silica aerogels and implications for material hydrophobicity and mechanical properties. J. Mater. Chem..

[96] Dong H., Brook M.A., Brennan J.D. (2005). A New Route to Monolithic Methylsilsesquioxanes:  Gelation Behavior of Methyltrimethoxysilane and Morphology of Resulting Methylsilsesquioxanes under One-Step and Two-Step Processing. Chem. Mater..

[97] Dong H., Brennan J.D. (2006). Macroporous Monolithic Methylsilsesquioxanes Prepared by a Two-Step Acid/Acid Processing Method. Chem. Mater..

[98] Dong H., Brennan J.D. (2006). Controlling the Morphology of Methylsilsesquioxane Monoliths Using a Two-Step Processing Method. Chem. Mater..

[99] Kanamori K., Aizawa M., Nakanishi K., Hanada T. (2007). New Transparent Methylsilsesquioxane Aerogels and Xerogels with Improved Mechanical Properties. Adv. Mater..

[100] Hayase G., Kanamori K., Nakanishi K. (2012). Structure and properties of polymethylsilsesquioxane aerogels synthesized with surfactant n-hexadecyltrimethylammonium chloride. Microporous Mesoporous Mater..

[101] Kanamori K., Aizawa M., Nakanishi K., Hanada T. (2008). Elastic organic–inorganic hybrid aerogels and xerogels. J. Sol-Gel Sci. Technol..

[102] Kanamori K., Nakanishi K., Hanada T. (2009). Sol-gel synthesis, porous structure, and mechanical property of polymethylsilsesquioxane aerogels. J. Ceram. Soc. Jpn..

[103] Kurahashi M., Kanamori K., Takeda K., Kaji H., Nakanishi K. (2012). Role of block copolymer surfactant on the pore formation in methylsilsesquioxane aerogel systems. RSC Adv..

[104] Wu X., Zhong K., Ding J., Shen X., Cui S., Zhong Y., Ma J., Chen X. (2020). Facile synthesis of flexible and hydrophobic polymethylsilsesquioxane based silica aerogel via the co-precursor method and ambient pressure drying technique. J. Non-Cryst. Solids.

[105] Huang S., Wu X., Li Z., Shi L., Zhang Y., Liu Q. (2020). Rapid synthesis and characterization of monolithic ambient pressure dried MTMS aerogels in pure water. J. Porous Mater..

[106] Hayase G., Kanamori K., Nakanishi K. (2011). New flexible aerogels and xerogels derived from methyltrimethoxysilane/dimethyldimethoxysilane co-precursors. J. Mater. Chem..

[107] Hayase G., Kanamori K., Nakanishi K., Hanada T. (2011). Synthesis of New Flexible Aerogels from Di- and Trifunctional Organosilanes. MRS Proc..

[108] Hayase G., Kanamori K., Fukuchi M., Kaji H., Nakanishi K. (2013). Facile Synthesis of Marshmallow-like Macroporous Gels Usable under Harsh Conditions for the Separation of Oil and Water. Angew. Chem..

[109] Tsuchiya R., Tanaka T., Hayase G., Kanamori K., Nakanishi K. (2015). Novel soft touch silicone beads from methyltrimethoxysilane and dimethyldimethoxysilane using easy aqueous solution reaction. J. Ceram. Soc. Jpn..

[110] Hayase G., Kanamori K., Hasegawa G., Maeno A., Kaji H., Nakanishi K. (2013). A superamphiphobic macroporous silicone monolith with marshmallow-like flexibility. Angew. Chem. Int. Ed. Engl..

[111] Hayase G., Nagayama S., Nonomura K., Kanamori K., Maeno A., Kaji H., Nakanishi K. (2018). Fabrication of hydrophobic polymethylsilsesquioxane aerogels by a surfactant-free method using alkoxysilane with ionic group. J. Asian Ceram. Soc..

[112] Durães L., Ochoa M., Portugal A., Duarte N., Dias J.P., Rocha N., Hernandez J. (2010). Tailored Silica Based Xerogels and Aerogels for Insulation in Space Environments. Adv. Sci. Technol..

[113] Ochoa M., Durães L., Beja A.M., Portugal A. (2011). Study of the suitability of silica based xerogels synthesized using ethyltrimethoxysilane and/or methyltrimethoxysilane precursors for aerospace applications. J. Sol-Gel Sci. Technol..

[114] Durães L., Ochoa M., Rocha N., Patrício R., Duarte N., Redondo V., Portugal A. (2012). Effect of the drying conditions on the microstructure of silica based xerogels and aerogels. J. Nanosci. Nanotechnol..

[115] Cai L., Shan G. (2015). Elastic silica aerogel using methyltrimethoxysilane precusor via ambient pressure drying. J. Porous Mater..

[116] Yang Z., Yu H., Li X., Ding H., Ji H. (2019). Hyperelastic and hydrophobic silica aerogels with enhanced compressive strength by using VTES/MTMS as precursors. J. Non-Cryst. Solids.

[117] Aravind P.R., Niemeyer P., Ratke L. (2013). Novel flexible aerogels derived from methyltrimethoxysilane/3-(2,3-epoxypropoxy)propyltrimethoxysilane co-precursor. Microporous Mesoporous Mater..

[118] Smitha V.S., Azeez P.M.A., Warrier K.G., Nair B.N., Hareesh U.N.S. (2018). Transparent and Hydrophobic MTMS/GPTMS Hybrid Aerogel Monoliths and Coatings by Sol-Gel Method: A Viable Remedy for Oil-Spill Cleanup. ChemistrySelect.

[119] Shajesh P., Smitha S., Aravind P.R., Warrier K.G. (2009). Synthesis, structure and properties of cross-linked R(SiO_1.5_)/SiO_2_ (R = 3-glycidoxypropyl) porous organic inorganic hybrid networks dried at ambient pressure. J. Colloid. Interface Sci..

[120] Ehgartner C.R., Grandl S., Feinle A., Husing N. (2017). Flexible organofunctional aerogels. Dalton Trans..

[121] Nadargi D.Y., Rao A.V. (2009). Methyltriethoxysilane: New precursor for synthesizing silica aerogels. J. Alloys Compd..

[122] Nadargi D.Y., Latthe S.S., Hirashima H., Rao A.V. (2009). Studies on rheological properties of methyltriethoxysilane (MTES) based flexible superhydrophobic silica aerogels. Microporous Mesoporous Mater..

[123] Aravind P.R., Soraru G.D. (2010). High surface area methyltriethoxysilane-derived aerogels by ambient pressure drying. J. Porous Mater..

[124] Cui S., Liu Y., Fan M.H., Cooper A.T., Lin B.L., Liu X.Y., Han G.F., Shen X.D. (2011). Temperature dependent microstructure of MTES modified hydrophobic silica aerogels. Mater. Lett..

[125] Li X., Yang Z., Li K., Zhao S., Fei Z., Zhang Z. (2019). A flexible silica aerogel with good thermal and acoustic insulation prepared via water solvent system. J. Sol-Gel Sci. Technol..

[126] Itagaki A., Nakanishi K., Hirao K. (2003). Phase Separation in Sol-Gel System Containing Mixture of 3- and 4-Functional Alkoxysilanes. J. Sol-Gel Sci. Technol..

[127] Shimizu T., Kanamori K., Maeno A., Kaji H., Doherty C.M., Falcaro P., Nakanishi K. (2016). Transparent, Highly Insulating Polyethyl- and Polyvinylsilsesquioxane Aerogels: Mechanical Improvements by Vulcanization for Ambient Pressure Drying. Chem. Mater..

[128] Wu Z., Zhang L., Li J., Zhao X., Yang C. (2018). Organic–inorganic hybridization for the synthesis of robust in situ hydrophobic polypropylsilsesquioxane aerogels with fast oil absorption properties. RSC Adv..

[129] Rao A.V., Kulkarni M.M., Pajonk G.M., Amalnerkar D.P., Seth T. (2003). Synthesis and Characterization of Hydrophobic Silica Aerogels Using Trimethylethoxysilane as a Co-Precursor. J. Sol-Gel Sci. Technol..

[130] Zhang L., Abbenhuis H.C., Yang Q., Wang Y.M., Magusin P.C., Mezari B., Van Santen R.A., Li C. (2007). Mesoporous organic-inorganic hybrid materials built using polyhedral oligomeric silsesquioxane blocks. Angew. Chem. Int. Ed. Engl..

[131] Qin Y., Ren H., Zhu F., Zhang L., Shang C., Wei Z., Luo M. (2011). Preparation of POSS-based organic–inorganic hybrid mesoporous materials networks through Schiff base chemistry. Eur. Polym. J..

[132] Bi Y.-T., Li Z.-J., Liu T.-S. (2014). Preparation and characterization of Octa(aminophenyl)silsesquioxane–aldehyde organic/inorganic hybrid aerogel. Eur. Polym. J..

[133] Duan Y., Jana S.C., Reinsel A.M., Lama B., Espe M.P. (2012). Surface modification and reinforcement of silica aerogels using polyhedral oligomeric silsesquioxanes. Langmuir.

[134] Wang L., Song G., Qiao X., Xiong G., Liu Y., Zhang J., Guo R., Chen G., Zhou Z., Li Q. (2019). Facile Fabrication of Flexible, Robust, and Superhydrophobic Hybrid Aerogel. Langmuir.

[135] Kanamori K., Nakanishi K., Hanada T. (2009). Spinodal decomposition in siloxane sol-gel systems in macroporous media. Soft Matter.

[136] Zu G., Shimizu T., Kanamori K., Zhu Y., Maeno A., Kaji H., Shen J., Nakanishi K. (2018). Transparent, Superflexible Doubly Cross-Linked Polyvinylpolymethylsiloxane Aerogel Superinsulators via Ambient Pressure Drying. ACS Nano.

[137] Zu G., Kanamori K., Shimizu T., Zhu Y., Maeno A., Kaji H., Nakanishi K., Shen J. (2018). Versatile Double-Cross-Linking Approach to Transparent, Machinable, Supercompressible, Highly Bendable Aerogel Thermal Superinsulators. Chem. Mater..

[138] Zu G., Kanamori K., Maeno A., Kaji H., Nakanishi K. (2018). Superflexible Multifunctional Polyvinylpolydimethylsiloxane-Based Aerogels as Efficient Absorbents, Thermal Superinsulators, and Strain Sensors. Angew. Chem. Int. Ed. Engl..

[139] Liu Z.J., Ran Y.Y., Xi J.N., Wang J. (2020). Polymeric hybrid aerogels and their biomedical applications. Soft Matter.

[140] Wang L., Feng J., Jiang Y., Li L., Feng J. (2019). Thermal conductivity of polyvinylpolymethylsiloxane aerogels with high specific surface area. RSC Adv..

[141] Wang L., Feng J., Jiang Y., Feng J. (2019). Polyvinylmethyldimethoxysilane reinforced methyltrimethoxysilane based silica aerogels for thermal insulation with super-high specific surface area. Mater. Lett..

[142] Rezaei S., Jalali A., Zolali A.M., Alshrah M., Karamikamkar S., Park C.B. (2019). Robust, ultra-insulative and transparent polyethylene-based hybrid silica aerogel with a novel non-particulate structure. J. Colloid. Interface Sci..

[143] Rezaei S., Zolali A.M., Jalali A., Park C.B. (2020). Novel and simple design of nanostructured, super-insulative and flexible hybrid silica aerogel with a new macromolecular polyether-based precursor. J. Colloid. Interface Sci..

[144] Shea K.J., Loy D.A. (2001). Bridged Polysilsesquioxanes. Molecular-Engineered Hybrid Organic−Inorganic Materials. Chem. Mater..

[145] Croissant J.G., Cattoen X., Durand J.O., Wong Chi Man M., Khashab N.M. (2016). Organosilica hybrid nanomaterials with a high organic content: Syntheses and applications of silsesquioxanes. Nanoscale.

[146] Stein A., Melde B.J., Schroden R.C. (2000). Hybrid Inorganic–Organic Mesoporous Silicates—Nanoscopic Reactors Coming of Age. Adv. Mater..

[147] Hatton B., Landskron K., Whitnall W., Perovic D., Ozin G.A. (2005). Past, Present, and Future of Periodic Mesoporous Organosilicas The PMOs. Acc. Chem. Res..

[148] Mizoshita N., Tani T., Inagaki S. (2011). Syntheses, properties and applications of periodic mesoporous organosilicas prepared from bridged organosilane precursors. Chem. Soc. Rev..

[149] Shea K.J., Moreau J., Loy D.A., Corriu R.J.P., Boury B. (2004). Bridged Polysilsesquioxanes. Molecular-Engineering Nanostructured Hybrid Organic-Inorganic Materials. Functional Hybrid Materials.

[150] Lindner E., Schneller T., Auer F., Mayer H.A. (1999). Chemistry in Interphases—A New Approach to Organometallic Syntheses and Catalysis. Angew. Chem. Int. Ed..

[151] Hatton B.D., Landskron K., Whitnall W., Perovic D.D., Ozin G.A. (2005). Spin-Coated Periodic Mesoporous Organosilica Thin Films—Towards a New Generation of Low-Dielectric-Constant Materials. Adv. Funct. Mater..

[152] Loy D.A., Shea K.J. (1995). Bridged Polysilsesquioxanes. Highly Porous Hybrid Organic-Inorganic Materials. Chem. Rev..

[153] Shea K.J., Loy D.A. (2001). A Mechanistic Investigation of Gelation. The Sol−Gel Polymerization of Precursors to Bridged Polysilsesquioxanes. Acc. Chem. Res..

[154] Dou C., Han L., Zhao S., Zhang H., Wang Y. (2011). Multi-Stimuli-Responsive Fluorescence Switching of a Donor−Acceptor π-Conjugated Compound. J. Phys. Chem. Lett..

[155] Shea K.J., Loy D.A., Webster O.W. (1989). Aryl-bridged polysilsesquioxanes--new microporous materials. Chem. Mater..

[156] Loy D.A., Jamison G.M., Baugher B.M., Russick E.M., Assink R.A., Prabakar S., Shea K.J. (1995). Alkylene-bridged polysilsesquioxane aerogels: Highly porous hybrid organic-inorganic materials. J. Non-Cryst. Solids.

[157] Loy D.A., Jamison G.M., Baugher B.M., Myers S.A., Assink R.A., Shea K.J. (1996). Sol−Gel Synthesis of Hybrid Organic−Inorganic Materials. Hexylene- and Phenylene-Bridged Polysiloxanes. Chem. Mater..

[158] Baugher B., Loy D.A., Assink R.A., Prabakar S., Shea K.J., Oviatt H. Porosity in hexylene-bridged polysilsesquioxanes: Effects of monomer concentration. Proceedings of the Fall meeting of the Materials Research Society (MRS).

[159] Boday D.J., Stover R.J., Muriithi B., Loy D.A. (2012). Mechanical properties of hexylene-and phenylene-bridged polysilsesquioxane aerogels and xerogels. J. Sol-Gel Sci. Technol..

[160] Loy D.A., Obrey-DeFriend K.A., Wilson Jr K.V., Minke M., Baugher B.M., Baugher C.R., Schneider D.A., Jamison G.M., Shea K.J. (2013). Influence of the alkoxide group, solvent, catalyst, and concentration on the gelation and porosity of hexylene-bridged polysilsesquioxanes. J. Non-Cryst. Solids.

[161] Aoki Y., Shimizu T., Kanamori K., Maeno A., Kaji H., Nakanishi K. (2016). Low-density, transparent aerogels and xerogels based on hexylene-bridged polysilsesquioxane with bendability. J. Sol-Gel Sci. Technol..

[162] Guo H., Nguyen B.N., McCorkle L.S., Shonkwiler B., Meador M.A.B. (2009). Elastic low density aerogels derived from bis[3-(triethoxysilyl)propyl]disulfide, tetramethylorthosilicate and vinyltrimethoxysilane via a two-step process. J. Mater. Chem..

[163] Wang Z., Dai Z., Wu J., Zhao N., Xu J. (2013). Vacuum-dried robust bridged silsesquioxane aerogels. Adv. Mater..

[164] Wang Z., Wang D., Qian Z., Guo J., Dong H., Zhao N., Xu J. (2015). Robust Superhydrophobic Bridged Silsesquioxane Aerogels tunable performances and their applications. ACS Appl. Mater. Interfaces.

[165] Yun S., Luo H., Gao Y. (2015). Low-density, hydrophobic, highly flexible ambient-pressure-dried monolithic bridged silsesquioxane aerogels. J. Mater. Chem. A.

[166] Wang Z., Dai Z., Zhao N., Zhang X., Dong H., Xu J. (2015). Mechanically robust aerogels derived from an amine-bridged silsesquioxane precursor. J. Sol-Gel Sci. Technol..

[167] Zhang Y., Wang J., Wei Y., Zhang X. (2017). Robust urethane-bridged silica aerogels available for water-carved aerosculptures. New J. Chem..

[168] Zou F., Yue P., Zheng X., Tang D., Fu W., Li Z. (2016). Robust and superhydrophobic thiourethane bridged polysilsesquioxane aerogels as potential thermal insulation materials. J. Mater. Chem. A.

[169] Shimizu T., Kanamori K., Maeno A., Kaji H., Nakanishi K. (2016). Transparent Ethylene-Bridged Polymethylsiloxane Aerogels and Xerogels with Improved Bending Flexibility. Langmuir.

[170] Shimizu T., Kanamori K., Maeno A., Kaji H., Doherty C.M., Nakanishi K. (2017). Transparent Ethenylene-Bridged Polymethylsiloxane Aerogels: Mechanical Flexibility and Strength and Availability for Addition Reaction. Langmuir.

[171] Schaefer D.W., Beaucage G., Loy D.A., Shea K.J., Lin J.S. (2004). Structure of Arylene-Bridged Polysilsesquioxane Xerogels and Aerogels. Chem. Mater..

[172] Wang Q., Mahadik D.B., Meti P., Gong Y.-D., Lee K.-Y., Park H.-H. (2020). Dioxybenzene-bridged hydrophobic silica aerogels with enhanced textural and mechanical properties. Microporous Mesoporous Mater..

[173] Mahadik D.B., Wang Q., Meti P., Lee K.-Y., Gong Y.-D., Park H.-H. (2020). Synthesis of multi-functional porous superhydrophobic trioxybenzene cross-linked silica aerogels with improved textural properties. Ceram. Int..

[174] Wei Y., Wang J., Zhang Y., Wang L., Zhang X. (2015). Autocatalytic synthesis of molecular-bridged silica aerogels with excellent absorption and super elasticity. RSC Adv..

[175] Chen D., Gao H., Jin Z., Wang J., Dong W., Huang X., Wang G. (2018). Vacuum-Dried Synthesis of Low-Density Hydrophobic Monolithic Bridged Silsesquioxane Aerogels for Oil/Water Separation: Effects of Acid Catalyst and Its Excellent Flexibility. ACS Appl. Nano Mater..

[176] Chen D., Gao H., Liu P., Huang P., Huang X. (2019). Directly ambient pressure dried robust bridged silsesquioxane and methylsiloxane aerogels: Effects of precursors and solvents. RSC Adv..

[177] Zhao J., Zeng G., Zou F., Jiang S., Chen Y., Wang H., Mu C., Tang X.Z. (2020). Bismaleimide bridged silsesquioxane aerogels with excellent heat resistance: Effect of sol–gel solvent polarity. Soft Matter.

[178] Koebel M., Rigacci A., Achard P. (2012). Aerogel-based thermal superinsulation: An overview. J. Sol-Gel Sci. Technol..

